# Brain-Computer Interfaces for Children With Complex Communication Needs and Limited Mobility: A Systematic Review

**DOI:** 10.3389/fnhum.2021.643294

**Published:** 2021-07-14

**Authors:** Silvia Orlandi, Sarah C. House, Petra Karlsson, Rami Saab, Tom Chau

**Affiliations:** ^1^Bloorview Research Institute, Holland Bloorview Kids Rehabilitation Hospital, Toronto, ON, Canada; ^2^Cerebral Palsy Alliance, The University of Sydney, Sydney, NSW, Australia; ^3^Institute of Biomedical Engineering (BME), University of Toronto, Toronto, ON, Canada

**Keywords:** brain-computer interface, children, youth, assistive technology, severe disability, communication, environmental control

## Abstract

Brain-computer interfaces (BCIs) represent a new frontier in the effort to maximize the ability of individuals with profound motor impairments to interact and communicate. While much literature points to BCIs' promise as an alternative access pathway, there have historically been few applications involving children and young adults with severe physical disabilities. As research is emerging in this sphere, this article aims to evaluate the current state of translating BCIs to the pediatric population. A systematic review was conducted using the Scopus, PubMed, and Ovid Medline databases. Studies of children and adolescents that reported BCI performance published in English in peer-reviewed journals between 2008 and May 2020 were included. Twelve publications were identified, providing strong evidence for continued research in pediatric BCIs. Research evidence was generally at multiple case study or exploratory study level, with modest sample sizes. Seven studies focused on BCIs for communication and five on mobility. Articles were categorized and grouped based on type of measurement (i.e., non-invasive and invasive), and the type of brain signal (i.e., sensory evoked potentials or movement-related potentials). Strengths and limitations of studies were identified and used to provide requirements for clinical translation of pediatric BCIs. This systematic review presents the state-of-the-art of pediatric BCIs focused on developing advanced technology to support children and youth with communication disabilities or limited manual ability. Despite a few research studies addressing the application of BCIs for communication and mobility in children, results are encouraging and future works should focus on customizable pediatric access technologies based on brain activity.

## Introduction

Technology is often exploited as a tool to support children affected by severe brain disorders or injury in their daily activities. These technologies are especially pertinent to children who are not capable of using speech to communicate or who are limited in motor skills and require mobility aids. Worldwide, only 1 in 10 people have access to assistive technology devices when required [World Health Organization (WHO), 2020] and in Canada 95% of 3,775,920 individuals living with a disability use at least one aid or device to assist movement, communication, learning, or daily activities of life (Berardi et al., 2020).

The need for novel assistive technology and techniques for neurorehabilitation effective for children is high (Mikołajewska and Mikołajewski, 2014). One of the most advanced technical solutions is the brain-computer interface (BCI). BCIs can be defined as a link between the brain and an extra-corporeal apparatus, whereby signals from the brain can directly control the external device entirely bypassing the peripheral nervous system (Wolpaw et al., 2000). BCIs utilize changes in brain activity occurring when we react to stimuli, perform specific mental tasks, or experience different psychological or emotional states. Non-invasive BCIs typically detect and utilize electromagnetic potentials directly related to ensemble neuronal firing, or the associated hemodynamic changes including regional changes in relative oxyhemoglobin and deoxyhemoglobin concentrations (Proulx et al., 2018; Schudlo and Chau, 2018; Sereshkeh et al., 2018, 2019) and changes in arterial blood flow velocities (Myrden et al., 2011, 2012; Goyal et al., 2016), due to neurovascular coupling. Clinically, BCIs enable brain-based control of communication aids and environmental technologies (Moghimi et al., 2013; Rupp et al., 2014), assist in diagnosis (De Venuto et al., 2016; Lech et al., 2019), and enhance rehabilitation therapies (Daly and Wolpaw, 2008; Pichiorri and Mattia, 2020).

A long-term objective of translational BCI research is providing a channel for communication and environmental control for people with severe and multiple physical disabilities who otherwise lack the means to interact with people and the environment around them (Wolpaw et al., 2002). Hence, BCI-based control has been explored for: computer cursors (Wolpaw et al., 2002; Wirth et al., 2020); virtual keyboards (Birbaumer et al., 1999; Thompson et al., 2014a; Hosni et al., 2019); augmentative and alternative access systems (Thompson et al., 2013, 2014b; Brumberg et al., 2018); prosthetic devices (McFarland and Wolpaw, 2008; Vilela and Hochberg, 2020); wheelchairs (Punsawad and Wongsawat, 2013; Yu et al., 2017); entertainment/gaming (Holz et al., 2013; Van de Laar et al., 2013; Cattan et al., 2020); Internet browsing (Mugler et al., 2010; Milsap et al., 2019); and painting (Münßinger et al., 2010; Zickler et al., 2013; Kübler and Botrel, 2019). Given these explorations, BCIs have potential to serve as an alternative access method for people with severe motor deficits (Huggins et al., 2014), who are not well-served by commercially available access solutions. Nonetheless, research on novel BCI solutions for target populations has been limited to laboratory settings (Fager et al., 2012; Wolpaw and Wolpaw, 2012; Guy et al., 2018) and able-bodied adults (Pires et al., 2011; Oken et al., 2018). A modest subset of BCI studies has recruited adults with disabilities, including: amyotrophic lateral sclerosis (Nijboer et al., 2008; Huggins et al., 2011; Oken et al., 2014); multiple sclerosis (Papatheodorou et al., 2019); brainstem stroke (Sellers et al., 2014); muscular dystrophy (Zickler et al., 2011); acquired brain injury (Huang et al., 2019) and cerebral palsy (CP) (Taherian et al., 2016).

The adult BCI focus is at least partially attributable to the relative ease of acquiring from this population, robust brain signals that can be well-characterized. While the findings of adult studies are promising, BCI algorithms optimized for adults cannot be directly applied to pediatric users due, in part, to age-related differences in the brain responses of interest (Volosyak et al., 2017; Manning et al., 2021). For example, compared to adults, children exhibit less language lateralization (Holland et al., 2001), attenuated movement-related cortical potentials (MRCPs) (Pangelinan et al., 2011), and greater attentional effects on the latencies of auditory evoked potentials (Choudhury et al., 2015). Well-established BCI tasks for adults, such as verbal fluency, a verbal working memory task that requires to recall words associated with a common criterion from memory (Schudlo and Chau, 2018), are not suitable for children without developmentally appropriate modifications (Gaillard et al., 2003; Schudlo and Chau, 2018). Children with congenital impairments may have atypical brain anatomy and functional organization that preclude the simple translation of time-honored BCI protocols, including those validated in adults with acquired impairments.

Developmental differences may also manifest behaviorally. Children may experience difficulties maintaining focus (Gavin and Davies, 2007; Kinney-Lang et al., 2020) and their brain signals can contain excessive movement artifacts (Bell and Wolfe, 2007). It is imperative that research expands beyond able-bodied adults and involves more end-users, including children, ensuring any new developments are optimized from an individual's perspective. Differences in brain structure, topography, cognitive processing pathways and psycho-behavioral predisposition ought to be considered (Weyand and Chau, 2017).

Mikołajewska and Mikołajewski (2014) published a mini-review of BCI applications in children identifying several issues unique to pediatric applications of BCI and a paucity of research thereof. Among these pediatric-specific challenges included the absence of guidelines for processing brain signals from children, heightened neural plasticity including evolving cortical organization and frequency content of signals, and child engagement considerations such as fear, comfort, and positioning (Mikołajewska and Mikołajewski, 2014). Notwithstanding these concerns, the need for pediatric BCI research remains high given the lack of viable access technologies for children and youth with severe and multiple disabilities (Myrden et al., 2014).

### Brain Computer Interfaces

BCI systems deploy either invasive or non-invasive signal acquisition modalities. Invasive BCIs monitor brain activity on a cortex's surface using electrocorticography (ECoG), within the gray matter using intracortical microelectrodes (Simeral et al., 2011) or in deep subcortical structures using depth electrodes (Krusienski and Shih, 2011; Herff et al., 2020). Non-invasive BCIs instead measure electrophysiological activity with electroencephalography (EEG) or magnetoencephalography (MEG), or hemodynamic activity using magnetic resonance imaging (MRI), near-infrared spectroscopy (NIRS) or transcranial Doppler ultrasound (Myrden et al., 2011, 2012). A third category, the hybrid BCI, is defined as systems using two or more measurement modalities such as NIRS-EEG and EEG-electrocardiogram (Pfurtscheller et al., 2010; Zephaniah and Kim, 2014) either simultaneously or sequentially.

BCIs can be categorized according to the paradigm invoked for eliciting machine-discernible brain signals. Reactive BCI paradigm elicits an event-related potential (ERP). Popular ERPs leveraged in BCIs include the P300, which is evoked by an oddball stimulus and characterized by a large positive deflection that occurs between 200–250 to 700–750 ms after stimulus onset (Amiri et al., 2013; He et al., 2020), and the steady-state visual evoked potential (SSVEP) and auditory steady-state response, wherein brain responses are evoked, respectively, by flickering lights or pure tones at specific frequencies. Active BCI paradigms elicit machine-discernible brain signals for BCI control *via* deliberate mental tasks such as motor imagery (MI), which involves the mental rehearsal of: a given movement (Rejer, 2012); mental arithmetic, music imagery (Weyand and Chau, 2015); spelling (Obermaier et al., 2003); covert speech (Birbaumer et al., 2010); observing pictures (Kushki et al., 2012); among others. Typical BCI taxonomies include passive BCIs that simply monitor the user's psychological state (Myrden and Chau, 2016, 2017).

**Figure 1:**
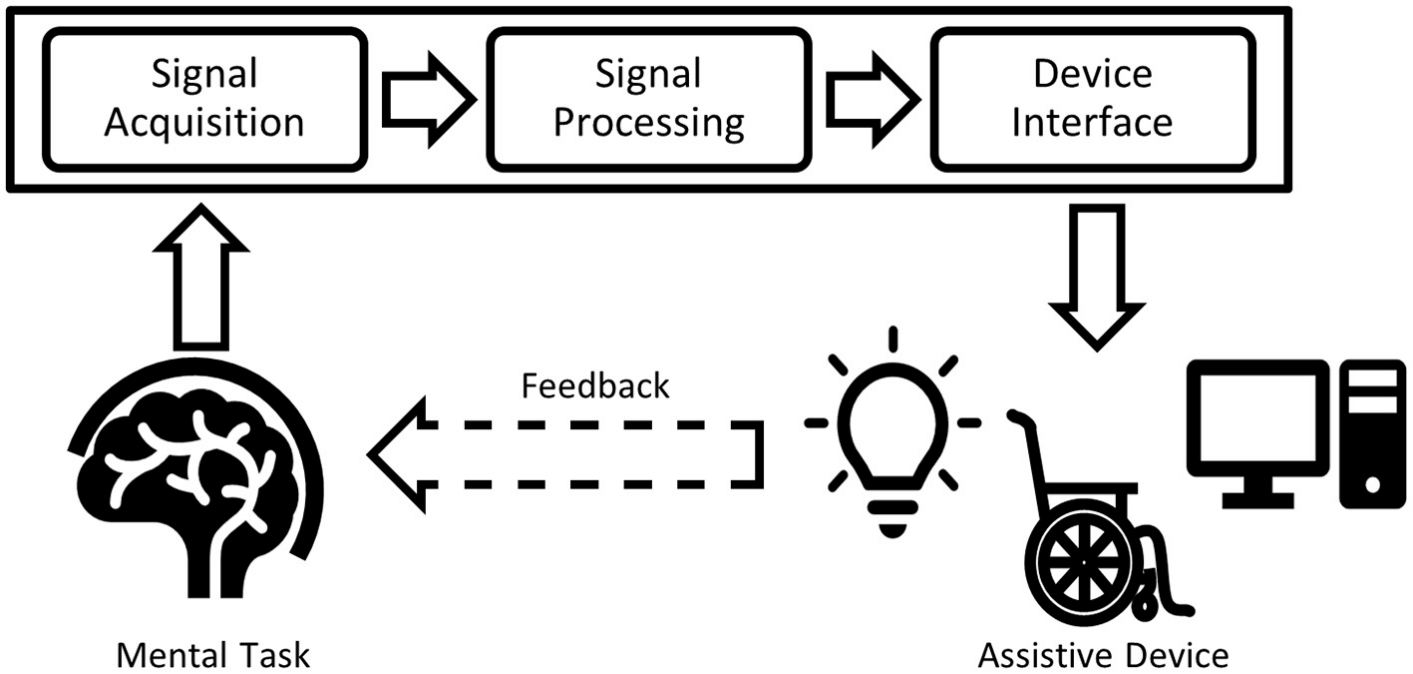
Typical BCI processing pipeline. The input signal acquired from the human brain is filtered (signal processing), classified and transferred to an output device (device interface), forming the BCI application.

Once acquired, signals generated by a BCI task are fed through a processing pipeline. Signal processing procedures for BCIs can be “offline” (retrospective) or “online” (suitable for real-time applications). A typical BCI processing pipeline (Bamdad et al., 2015) for communication and mobility is depicted in Figure 1. Typical pipeline elements include algorithms to suppress artifacts, extract features, and classify the signals. Pipeline outputs are then used to control an assistive device that supports communication or mobility.

### Objectives and Research Question

This article appraises the pediatric BCI literature systematically, considering specific inclusion criteria and highlighting the current information processing methods applied to pediatric brain signals. Through this systematic review, we set out to address two research questions: (1) What is the state of science in applying BCIs to support communication and manual ability in the pediatric population?; (2) What is current knowledge about the necessary considerations to render BCIs suitable for children?

## Methods

### Study Design

This systematic review included all levels of research evidence and aimed to integrate best practice systematic review methodology, the Preferred Reporting Items for Systematic Reviews and Meta-Analyses (PRISMA) guidelines (Moher et al., 2009).

### Search Strategy

#### Identification Process

Based on a preliminary search-string with the PubMed database, the syntax was developed for the search across three databases during May 2020: Scopus, PubMed, and Ovid. The SPIDER (Sample, Phenomenon of Interest, Design, Evaluation, Research type) tool was used to structure the search related to the research questions (Cooke et al., 2012). Electronic database searches were performed using the following key-terms related to “Sample:” *pediatric* or *pediatric*; or *child* or *children*; or *youth(s)* or *adolescent(s)* or *teen(s)* or *teenager(s)*. These were combined with the following “Phenomenon of Interest” terms: *BCI* or *brain computer interface* or *brain-computer interface* or *brain-machine interface* or *brain machine interface* or *mind machine interface* or *direct neural interface* or *neural control interface*. The search strategy did not specify design, evaluation, or research type in order to capture all potentially relevant articles. These terms were considered in the inclusion and exclusion criteria. After retrieving studies from the searches, duplicates were removed and the paper titles, abstracts, and associated meta-data were compiled into a single table for further review.

#### Screening Process: Inclusion and Exclusion Criteria

All research within Oxford levels of evidence I–IV (Howick et al., 2011), including case studies and single-case experimental design studies reporting objective outcome measures were eligible for inclusion, if they: (1) reported in full text; (2) were published in English in peer-reviewed journals between January 2008 and May 2020; (3) included children and adolescents, under the age of 19 (Sawyer et al., 2018), using BCIs; (4) described the design of the protocol used for data collection (“Design”); (5) measured outcomes related to the performance of the BCI (“Evaluation”); and (6) included quantitative methods (“Research type”). Studies that presented only aggregate results from participating adults and children were excluded. Those studies that related to the general diagnosis of brain disorders or diseases were excluded. Publications related to passive BCI without a final goal of developing assistive technology devices were excluded. Gray literature and unpublished works were not eligible for inclusion. Strictly qualitative research, book chapters, review articles, and conference publications were excluded.

#### Eligibility Process: Study Selection, Data Collection Process, and Synthesis of Results

Two of the five authors (SO and SCH) conducted the search across the databases and produced a list of articles based on the title and abstract according to the inclusion criteria. A two-step procedure was carried out independently by four authors (SO, SCH, PK, RS) to identify articles for inclusion. The first step involved screening titles and abstracts for potential eligibility and, thereafter, screening the full text of potentially eligible articles. Four authors independently completed data extraction. An almost perfect level of agreement was obtained for title and abstract screening (Cohen's kappa coefficient, *k* = 0.96, percentage of agreement 98%). After a full-text review of the eligible papers, articles were excluded for any of the following reasons: the performance was reported for a heterogeneous group composed of adults and children without a two-group comparison (e.g., only averaged accuracy was reported, or children and adults' classification performance was not distinguishable); BCIs were not developed for pediatric participants; BCIs developed for adults but included a limited number of children (only one or two adolescents not sufficient for a two-group comparison); only adult participants were included in the study; results were not reported in terms of BCI performance; the study was not related to BCIs; passive BCIs were applied; the study did not include participants' data; participants' ages were not reported. Twelve articles remained eligible for further review. Twelve articles remained eligible for further review.

### Data Extraction and Analysis

For each eligible study, the following data were extracted: number of participants and their ages; study design and data acquisition protocol; signal features; classifier; and performance metrics. It was not appropriate to conduct a meta-analysis or any statistical analyses of the results due to the small number and heterogeneity of the included studies. Instead, key findings were summarized and presented narratively clustering the selected full-text papers into two sub-groups based on the functional activities identified (communication or manual ability) and the type of measurement applied (non-invasive or invasive). No additional articles were found by consulting the references of the included full-text articles.

### Quality Appraisal and Risk of Bias

Considering the heterogeneity of the 12 articles, the “QualSyst” quality assessment tool (Alberta Heritage Foundation for Medical Research) was used to gauge the quality of the overall body of evidence (Kmet et al., 2004). We applied a 14-criteria checklist for quantitative studies, where raters scored each criterion as being fully (2 points), partially (1 point), or not (0 points) fulfilled. A summary score was calculated for each paper as the sum of the scores across all applicable criteria and expressed as a percentage of the total possible score. When a specific criterion was not applicable to a given study, the criterion was omitted from the calculation of the summary score. Two reviewers (SO and SCH) independently assessed the quality (inter-rater reliability, *k* = 0.77 and 86% level of agreement) and the risk of bias for all the included studies. The sample sizes of the multiple-case-study articles were reported in terms of the number of pediatric participants recruited in the studies. Adult participants were not included in Tables 1–6. To further elucidate the overall quality of the evidence, each of the included articles received a quality grade as: limited (score of ≤50%); adequate (>50 and ≤70%); good (>70 and ≤80%); or strong (>80%) (Lee et al., 2008). Discrepancies were discussed between the two reviewers and consensus was reached. The risk of bias was identified for each study by two authors (SO and SCH) using the Agency for Healthcare Research and Quality (AHRQ) criteria (Viswanathan et al., 2017). The risk of bias was assessed through the evaluation and discussion of each article in terms of selection, performance, attrition, detection, and reporting (inter-rater reliability, *k* = 0.94 and 95% level of agreement). Responses for each criterion were scored as “low risk,” “high risk,” “unclear,” and “not applicable.” Low risk of bias was assumed when studies met all the risk-of-bias criteria, medium risk of bias if at least one of the risk-of-bias criteria was not met and high risk of bias if three or more risk-of-bias criteria were not fulfilled. An unknown risk of bias was considered as high risk.

**Table 1:** Research articles on pediatric non-invasive BCIs: study objectives and data collection details.

**References**	**Study objective**	**Sample size [females]**	**Age (years)**	**Diagnosis**	**Applications**	**BCI paradigm**	**Mode of operation**	**Signal type**	**Data acquisition**	**Task and sessions**
Ehlers et al. (2012)	Examine age-related performance differences on an SSVEP-BCI	*N* = 51 [31] Pediatric Group 1: *N* = 11 [6] Group 2: *N* = 12 [9] Group 3: *N* = 14 [3]	6–33 Pediatric Avg. 6.73 Avg. 8.08 Avg. 9.86	TD	Mouse control/ spelling	SSVEP # stimuli: 3 Low: 7–11 Hz Medium: 13–17 Hz High: 30, 32, 34, 36, 38 Hz	Synch	EEG	***Location:*** parietal and occipital (PZ, PO3, PO4, O1, OZ, O2, O9, O10) ***# Channels**: 8* ***Hardware and Software:** -*Ag/Ag-Cl EEG electrodes -Wet system *-*BCI2000 -C++ (Bremen BCI)	***Task**:* cursor control to complete a spelling task ***# Sessions***: 1 ***Session duration**:* 45 min ***Task duration***: 2 min per run (6 words and at least 20 commands per word)
Norton et al. (2018)	Compare the performance of 9–11-year-old children using SSVEP-based BCI to adults	*N* = 26 [n/a] Pediatric *N* = 15 [n/a]	9–68 Pediatric 9–11	TD	Graphical interface comprising three white circle targets	SSVEP # stimuli: 3 [6.2, 7.7, 10 Hz]	Synch	EEG	***Location:*** PO3, POZ, PO4, O1, OZ, O2 ***# Channels:*** 6 ***Hardware and Software:*** -Tin electrodes -Wet system -BCI2000	***Task:*** focus visual attention on one of three white circle targets **# Sessions**: 1 ***Session duration:*** 5 trials/stimulus (15 trials total) for training, 20 trials/stimulus (60 trials total) for testing. ***Task duration***: 5 s
Beveridge et al. (2017)	Evaluate mVEP paradigm for BCI-controlled video game	Pediatric *N* = 15 [4]	Pediatric 13–16	TD	Neurogaming	mVEP # stimuli: 5	Synch	EEG	***Location:*** Cz, TP7, CPz, TP8, P7, P3, Pz, P4, P8, O1, Oz, and O2 ***# Channels:*** 12 ***Hardware and Software***: -g.LADYbird -g.BSamp and g.GAMMAbox -MATLAB® -Unity 3D	***Task:*** 3D car-racing video game ***# Sessions:*** 1 **Session duration:** 1 h ***Task duration:*** 1,000 ms to activate 5 stimuli (300 trials per calibration and 60 per testing)
Beveridge et al. (2019)	Study trade-off between accuracy of control and gameplay speed using an mVEP BCI	*N* = 48 [10] Pediatric *N* = 15 [4]	13–40 Pediatric 13–16	Pediatric data is pulled from Beveridge et al. (2017) to compare to newly collected adult data. Adult protocol differed slightly from pediatric protocol (e.g., slow medium and fast lap rather than 3 slow laps + compare experienced vs. naive adults).						
Vařeka (2020)	Compare CNN with baseline classifiers using large subject P300 BCI dataset	Pediatric *N* = 250 [112]	7–17	No identifying physical symptoms were asked or recorded	Guess the number game	P300 # stimuli: 1-9 flashings	Synch	EEG	***Location:*** Fz, Cz, Pz ***# Channels:*** 3 ***Hardware and Software:*** -BrainVision standard V-Amp -Neurobehavioural Systems Inc. -BrainVision Recorder -MATLAB®	***Task:*** P300 ***# Sessions:*** 1 ***Session duration:*** ***Task duration:*** 1,000 ms (532 trials)
Taherian et al. (2017)	Employ a commercial EEG based BCI with people with CP	*N* = 8 [4] Pediatric *N* = 5 [2]	7–43 Pediatric P1: 17 P2: 17 P3: 7 P4: 9 P5: 9	Spastic quadriplegic CP	Puzzle games	MI-ERD	Synch	EEG	***Location*****:** unknown number—includes C3 and C4 ***# Channels*****:** 14 ***Hardware and Software:*** *-*Emotiv EPOC BCI headset -Saline felt electrodes -Emotiv software	***Task**:* imagined arm movements associated with the ability to move a virtual cube ***# Sessions***: 5–7 ***Session duration***: 30 min ***Task duration***: 16–55 8 s trials
Jochumsen et al. (2018)	Movement intention detection in adolescents with CP from single-trial EEG	Pediatric *N* = 8 [1]	11–17 P1: 16 P2: 15 P3: 11 P4: 15 P5: 13 P6: 15 P7: 17 P8: 16	Hemiplegia or diplegia CP with GMFCS of I-V	Neurorehabilitation	Movement preparation—MRCP/ dorsiflexions of the ankle joint	Asynch (self-paced)	EEG	***Location:*** F3, FZ, F4, C3, CZ, C4, P3, PZ, P4 ***# Channels:*** 9 ***Hardware and Software:*** -Neuroscan EEG amplifiers -Wet system -EMG for movement detection -MATLAB®	***Task:*** dorsiflexion of the ankle joint ***# Sessions:*** 1 ***Session duration:*** 15 min—avg. 65 ± 18 movements performed per participant ***Task duration:*** 4–6 s
Zhang et al. (2019)	Evaluate if children can use simple BCIs	Pediatric *N* = 26 [7]	6–18 P1–2: 6 P3: 8 P4: 9 P5–7: 10 P8–9: 11 P10–11: 12 P12–14: 13 P15–16: 14 P17: 15 P18–19: 16 P20–22:17 P23–26: 18	TD	Mouse control and remote-controlled car	MI and goal-oriented thinking—ERD	Synch	EEG	***Location**:* AF3, AF4, F3, F4, F7, F8, FC5, FC6, P7, P8, T7, T8, O1, O2 ***# Channels:*** 14 ***Hardware and Software*****:** -Emotiv EPOC BCI headset -Saline felt electrodes -Emotiv software	***Tasks:*** imagine opening and closing both hands, “goal-oriented thoughts,” and rest **# Sessions**: 2 ***Session duration:*** <1 h ***Task duration***: eight 8 s trials per task for training; 10–20 s trials with 5 s rest for testing

*CP, cerebral palsy; avg., average; MI, motor imagery; N, number of participants; P#, pediatric participant number; SSVEP, steady state visual evoked potential; synch, synchronous; asynch, asynchronous; TD, typically developing; CNN, convolutional neural network; mVEP, motion-onset visual evoked potential; EEG, electroencephalography; EMG, electromyography; ERD, event-related desynchronization; MRCP, movement-related cortical potential; GMFCS, gross motor function classification system; n/a, not applicable*.

**Table 2:** Research articles on pediatric non-invasive BCIs: signal processing techniques and results (only for pediatric age).

**References**	**Online/offline/# of classes**	**Signal processing and features**	**Classifier or analysis**	**Results**
Ehlers et al. (2012)	***Online only*** 5 classes: 5 visual stimulation frequency targets in low, medium, or high frequency range Chance level: n/a	***Fs***: 2,048 Hz ***Filtering***: High-pass (fc = 0.1 Hz) Low-pass (fc = 552.96 Hz) ***Features:*** Minimum energy combination spatial filter	***Classifier:*** Bremen-BCI ***Outcome Measure:*** (i) Accuracy	***Offline accuracy:*** n/a ***Online accuracy:*** Low-frequency stimulation Group 1: 40%; Group 2: 50%; Group 3: 50% Medium-frequency stimulation Group 1: 55%; Group 2: 50%; Group 3: 75% High-frequency stimulation Group 1: 38%; Group 2: 45%; Group 3: 55% ***Additional measures:*** n/a
Norton et al. (2018)	***Offline and Online*** 3 classes: 3 SSVEP targets Chance level: n/a	***Fs***: 128 Hz ***Filtering***: Bandpass 1–30 Hz ***Features:*** (i) Threshold (ii) Window-length	***Classifier:*** Canonical correlation analysis ***Outcome Measure:*** (i) Classification accuracy (ii) Latency (iii) Nykopp bitrate	***Offline accuracy:*** 11 of 14 children exceeded the threshold of success: 40–100% ***Online accuracy:*** P: 79% ***Additional measures:*** Latency: 2.106 s Nykopp bitrate: 0.5 bits s^−1^
Beveridge et al. (2017)	***Offline*** 5 classes: 5 target locations for mVEP and 2 classes: Leave one out cross validation among the 5 targets ***Online*** 5 target locations classified using target vs. non-target binary classification Chance level: 20% (0 bpm) for 5 class-classifier 50% for 2 class-classifier (theoretical chance level)	***Fs:*** 250 Hz, resampled to 20 Hz ***Filtering:*** Baseline-corrected, Low-pass filter (fc = 10 Hz) ***Features:*** (i) mVEP components (e.g., P100, N200, and P300): data averaged over 5 trials (12 feature vectors per stimulus)	***Classifier:*** LDA ***Outcome Measure:*** (i) Classification accuracy (ii) ITR	***Offline accuracy (LOOCV & 5-class):*** P1: 84.58 & 76.67 P9: 94.79 & 98.33 P2: 94.58 & 96.67 P10: 78.75 & 71.67 P3: 84.79 & 81.67 P11: 90.42 & 91.67 P4: 83.54 & 70.00 P12: 77.50 & 68.33 P5: 86.46 & 85.00 P13: 88.75 & 85.00 P6: 77.29 & 76.67 P14: 82.29 & 75.00 P7: 88.96 & 85.00 P15: 72.92 & 70.00 P8: 91.88 & 81.67 Mean: 85.17 & 80.89 ***Online accuracy:*** P1: 54%^*^ P9: 75%^*^ P2: 88%^*^ P10: 51%^*^ P3: 45%^*^ P11: 58%^*^ P4: 65%^*^ P12: 60%^*^ P5: 83%^*^ P13: 85%^*^ P6: 61%^*^ P14: 82%^*^ P7: 82%^*^ P15: 40%^*^ P8: 92%^*^ Mean: 68%^**^ ***Additional measures:*** ITR P1: 4 bpm^*^ P9: 13 bpm^*^ P2: 19 bpm^*^ P10: 4 bpm^*^ P3: 3 bpm^*^ P11: 6 bpm^*^ P4: 8 bpm^*^ P12: 7 bpm^*^ P5: 16 bpm^*^ P13: 17 bpm^*^ P6: 7 bpm^*^ P14: 16 bpm^*^ P7: 16 bpm^*^ P15: 2 bpm^*^ P8: 21 bpm^*^ Mean: 11 bpm^**^
Beveridge et al. (2019)	***Offline*** 5 classes: 5 target locations for mVEP and 2 classes: LOOCV among the 5 targets ***Online*** 5 target locations classified using target vs. non-target binary classification Chance level: 20% (0 bpm) for 5 class-classifier 50% for 2 class-classifier (theoretical chance level)	***Fs:*** 250 Hz, resampled to 20 Hz ***Filtering:*** Baseline-corrected, Low-pass filter (fc = 10 Hz) ***Features:*** (i) mVEP components (e.g., P100, N200, and P300): data averaged over 5 trials (12 feature vectors per stimulus)	***Classifier:*** LDA ***Outcome Measure:*** (i) Classification accuracy (ii) ITR (iii) mVEP latency (iv) mVEP amplitude	-Same results as Beveridge et al. (2017) -Online results showed that BCI naïve adults achieved higher accuracies than BCI naïve children (the difference is not always statistically significant)
Vařeka (2020)	***Offline only*** 10 classes: 10 P300 targets Chance level: n/a	***Fs:*** 1,000 Hz ***Filtering:*** Baseline-corrected, amplitude threshold 100 μV ***Features:*** (i) Averaged time intervals and feature scaled to zero mean and unit variance	***Classifier:*** LDA, SVM, and CNN in leave one out cross validation ***Outcome Measure:*** (i) Accuracy (ii) Precision (iii) Recall (iv) AUC	***Offline accuracy:*** Single-trial classification accuracy 62–64% Accuracy with trial averaging 76–79% ***Online accuracy:*** n/a ***Additional measures:*** (i) Single-trial classification (ii) Precision-−61.5–63.5%^***^ (iii) Recall-−60.5–67.5%^***^ (iv) AUC-−62–66%^***^all tested models achieved comparable classification results
Taherian et al. (2017)	***Online only*** 2 classes: Left and right arm motor imagery Chance level: n/a	***Fs:*** n/a ***Filtering:*** Proprietary Emotiv software ***Features:*** Proprietary Emotiv software (Cognitiv suite)—ERD	***Classifier:*** Emotiv classifier—proprietary output from Emotiv Software Development Kit ***Outcome Measure:*** (i) peak performance score	***Offline accuracy:*** n/a ***Online accuracy:*** n/a ***Additional measures:*** Peak performance score for left and right arm
Jochumsen et al. (2018)	***Offline only*** 2 classes: idle vs. movement-related activity Chance level: 60–65%	***Fs**:*1,000 Hz ***Filtering***: 4th order zero phase shift Butterworth bandpass 0.1–45 Hz, baseline correction ***Features**:* (i) Mean amplitudes (ii) Absolute band power (iii) Template matching (iv) All features combined	***Classifier**:* Random forest classifier in LOOCV ***Outcome Measure:*** (i) Classification accuracy	***Offline accuracy:*** 75–85% ***Online accuracy:*** n/a ***Additional measures:*** n/a
Zhang et al. (2019)	***Online only*** 2 classes: MI/goal-oriented thought and rest Chance level: 70% (0.40 Cohen's Kappa)	***Fs:*** 2,048 Hz resampled to 128 Hz ***Filtering:*** Proprietary Emotiv software ***Features:*** Proprietary Emotiv software—ERD	***Classifier:*** Emotiv classifier—PNN and RBF ***Outcome Measure:*** (i) Cohen's kappa	***Offline accuracy:*** n/a ***Online accuracy:*** n/a ***Additional measures:*** Average Kappa score of 0.46, range of 0.025–0.9

*Fs, sampling frequency; fc, cut-off frequency; SSVEP, steady state visually evoked potential; mVEP, motion-onset visual evoked potential; MI, motor imagery; ITR, information transfer rate; LOOCV, leave-one-out cross-validation; CNN, convolutional neural network; LDA, linear discriminant analysis; SVM, support vector machine; AUC, area under the ROC curve*;

* *Averaged across all 3 laps (estimated from bar graph)*;

** *Averaged across all 3 laps*;

*** *Estimated from bar graph-range includes achieved averages for all three classifier results; ERD, event-related desynchronization; PNN, probabilistic neural network; RBF, radial basis function; bpm, bits per minute; P#, pediatric participant number; n/a, not applicable*.

**Table 3:** Research articles on pediatric invasive BCIs: Study objectives and data collection details.

**References**	**Study objective**	**Sample size [females]**	**Age (years)**	**Diagnosis**	**Applications**	**BCI paradigm**	**Mode of operation**	**Signal type**	**Data acquisition**	**Task and sessions**
Sanchez et al. (2008)	Present techniques to spatially localize motor potentials	Pediatric *N* = 2 [2]	14–15 P1: 14 P2: 15	Intractable epilepsy	Neuroprosthetics	Arm reaching and pointing	Synch	ECoG	***Location:*** sensorimotor cortex ***# Channels:*** 36 and 32 ***Hardware and Software:*** -MATLAB®	***Task:*** arm reaching and pointing ***# Sessions:*** 1 ***Session duration:*** 6 task repetitions ***Task duration:*** 5 s
Breshears et al. (2011)	Decodable nature of pediatric brain signals for the purpose of neuroprosthetic control	*N* = 11 [n/a] Pediatric *N* = 6 [1]	9–46 Pediatric 9–15 P1: 15 P2: 11 P3: 15 P4: 9 P5: 12 P6: 13	Intractable epilepsy	Neuroprosthetics/mouse control	MI or motor execution (hand opening/closing, tongue protrusions, phoneme articulation)	Synch	ECoG	***Location**:* motor, temporal, and prefrontal areas, depending on the patient ***# Channels*****:** 48 or 64 ***Hardware and Software**:* -AdTech electrode arrays - g.tec amplifier -BCI2000 -MATLAB®	***Task**:* move a cursor on a screen along one-dimension using motor execution or imagined movement **# Sessions**: 1 ***Session duration***: 10–37 min ***Task duration***: 2–3 s
Pistohl et al. (2012)	ECoG signal decoding for hand configurations in an everyday environment	Pediatric *N* = 3 [3]	14–16 P1: 14 P2: 16 P3: 15	Epilepsy	Neuroprosthetics/reach-to-grasp	Motor execution	Asynch (self-paced)	ECoG	***Location:*** electrodes residing over hand-arm motor cortex as identified through anatomical location and electrical stimulation **# Channels**: 48 or 64 ***Hardware and Software:*** -IT-Med clinical EEG-System	***Task**:* reach-to-grasp movements (self-paced and largely self-chosen movements) ***# Sessions**: 1* ***Session duration: –*** ***Time of analyzed data:*** P1: 32 min (303 grasps) P2: 35.3 min (338 grasps) P3: 25.4 min (320 grasps) ***Task duration***: 60 ms per grasp
Pistohl et al. (2013)	Time of grasps from human ECoG recording from the motor cortex during a sequence of natural and continuous reach-to-grasp movements	Pediatric *N* = 3 [3]	14–16 P1: 14 P2: 16 P3: 15	Same as Pistohl et al. (2012) as the participants and experimental paradigm is the same.						

*N, number of participants; ECoG, electrocorticography; P#, pediatric participant number; MI, motor imagery; synch, synchronous; asynch, asynchronous; n/a, not applicable*.

**Table 4:** Research articles on pediatric invasive BCIs: signal processing techniques and results (only for pediatric age).

**References**	**Online/offline/# of classes**	**Signal processing and features**	**Classifier/outcome measures**	**Results**
Sanchez et al. (2008)	n/a Chance level: n/a	***Fs:*** 381.5 Hz ***Filtering:*** FIR filter (1–6 kHz) ***Features:*** (i) Equiripple FIR filter: 1–60 Hz, 60–100 Hz, 100–300 Hz, 300 Hz−6 kHz (ii) FIR filter topology trained using the Wiener solution	***Classifier:*** n/a ***Outcome Measure:*** (i) Pearson's r	***Offline accuracy:*** n/a ***Online accuracy:*** n/a ***Additional measures:*** Pearson's *r* for X-position; Y-position P1: 0.39 ± 0.26; 0.48 ± 0.27 P2: 0.42 ± 0.26; 0.45 ± 0.25 Highest *r* achieved with 300 Hz−6 kHz feature
Breshears et al. (2011)	***Online only*** 2 classes: Imagined or performed motor movement vs. rest Chance level: 50% (theoretical chance level)	***Fs**:* 1,200 Hz ***Filtering:*** Autoregressive spectral coefficients in 2 Hz frequency bins from 0 to 250 Hz for each electrode ***Features:*** (i) Spectral power of filtered frequency bins (ii) Spectral power of electrodes	***Classifier**:* Real-time translational algorithm based on the weighted linear summation of the identified features (showing power increases were assigned positive weights, or power decreases were assigned negative weights) ***Outcome Measure:*** (i) Accuracy for each action	***Offline accuracy:*** n/a ***Online accuracy:*** P1: 70.8–99.0% P2: 72.7–77.4% P3: 82.7–85.1% P4: 75.0–100% P5: 88.8 % P6: 93.3 % ***Additional measures:*** n/a
Pistohl et al. (2012)	***Offline*** 2 classes: precision grip, whole-hand grip 10-fold cross-validation (20 repetitions) Chance level: 50% (theoretical chance level)	***Fs:*** 256 Hz ***Filtering:*** Re-referenced to common average, average voltage subtracted, normalized voltage, low pass filtered component (fc ~5 Hz) ***Features:*** (i) Signal components	***Classifier:*** rLDA ***Outcome Measure:*** (i) Decoding accuracy (ii) Temporal evolution of grasp discriminability	***Offline accuracy:*** P1: 97% P2: 84% P3: 96% ***Online accuracy:*** n/a ***Additional measures:*** Temporal evolution of grasp discriminability: 0.2 s
Pistohl et al. (2013)	***Offline only*** 2 classes: grasp and no grasp Chance level: n/a	***Fs***: 256 or 1,024 Hz***Filtering***:LFC: 2nd order Savitzky-Golay filter (window length: 250 ms) ***Features:*** (i) LFC (ii) Frequency band amplitudes within consecutive bands of 4 Hz width from 0 to 128 Hz. Band pass filtering by 4th order elliptic digital filter design	***Classifier**:* rLDA in 10-fold cross-validation ***Outcome Measures:*** (i) True positive ratio (TPR) (ii) False positive ratio (FPR) (iii) False positive min^−1^ (FP-rate)	***Offline accuracy:*** After 0.25 s (TPR/FPR/FP-rate): P1: 0.75/0.26/2.5 P2: 0.50/0.36/2.7 P3: 0.75/0.25/3.1 After 0.50 s (TPR/FPR/FP-rate): P1: 0.92/0.10/0.9 P2: 0.69/0.12/0.9 P3: 0.91/0.08/1.0 After 0.75 s (TPR/FPR/FP-rate): P1: 0.97/0.05/0.4 P2: 0.74/0.05/0.4 P3: 0.96/0.03/0.4 ***Online accuracy:*** n/a ***Additional measures:*** n/a

*Fs, sampling frequency; fc, cut-off frequency; rLDA, regularized linear discriminant analysis; FPR, false positive ratio; TPR, true positive ratio; FP-rate, false positive rate; P#, pediatric participant number; LFC, low-pass filtered component; FIR, finite impulse response; n/a, not applicable*.

**Table 5:** QualSyst scores for quantitative papers.

**References**	**QUALSYST criteria (quantitative)**														**Score (%)**	**Quality grade**
	**Question/objective sufficiently described**	**Study design evident and appropriate**	**Method of subject/comparison group selection or source of information/input variable described and appropriate**	**Subject characteristic sufficiently described**	**If interventional and random allocation was possible, was it described?**	**If interventional and blinding of investigators was possible, was it reported?**	**If interventional and blinding of subjects was possible, was it reported?**	**Outcomes and (if applicable) exposure measures well-defined and robust to measurement/misclassification bias? Means of assessment reported?**	**Sample size appropriate (pediatric population)**	**Analytic methods described/justified and appropriate**	**Some estimate of variance is reporter for the main results**	**Controlled for confounding**	**Results reported in sufficient detail**	**Conclusions supported by the results**		
Sanchez et al. (2008)	2	2	1	2	N/A	N/A	N/A	1	0	1	1	0	2	2	70	Adequate
Breshears et al. (2011)	2	2	1	2	N/A	N/A	N/A	2	0	1	0	N/A	1	1	60	Adequate
Ehlers et al. (2012)	2	2	1	2	N/A	N/A	N/A	1	0	2	1	1	2	2	73	Good
Pistohl et al. (2012)	2	2	1	2	N/A	N/A	N/A	2	0	2	0	0	2	2	75	Good
Pistohl et al. (2013)	1	1	1	2	N/A	N/A	N/A	2	0	2	1	N/A	2	2	70	Adequate
Beveridge et al. (2017)	1	2	1	2	N/A	N/A	N/A	1	0	1	0	1	1	2	60	Adequate
Taherian et al. (2017)	2	2	1	2	N/A	N/A	N/A	0	0	0	0	N/A	0	2	45	Limited
Jochumsen et al. (2018)	2	2	1	2	N/A	N/A	N/A	2	0	2	1	N/A	2	2	80	Good
Norton et al. (2018)	2	2	1	1	N/A	N/A	N/A	2	1	1	1	1	1	2	68	Adequate
Beveridge et al. (2019)	1	2	1	2	N/A	N/A	N/A	1	0	1	0	1	1	2	60	Adequate
Zhang et al. (2019)	2	2	2	1	N/A	N/A	N/A	2	1	2	2	2	2	2	91	Strong
Vařeka (2020)	2	2	1	1	N/A	N/A	N/A	1	2	2	2	1	2	2	90	Strong

*Criteria were scored either 2 or 1 or 0 (2 = yes, 1 = partial, 0 = no) or if the criteria were not applicable to the paper it was scored N/A (not applicable). To make them comparative, overall scores are presented as a %. Quality grade: limited (score of ≤50%), adequate (>50% and ≤70%), good (>70% and ≤80%), strong (>80%) (Lee et al., 2008)*.

**Table 6:** Risk of bias.

**References**	**Source of bias**					
	**Selection bias**	**Performance bias**	**Attrition bias**	**Detection bias**	**Reporting bias**	**Overall bias**
Sanchez et al. (2008)	+	–	?	+	–	High
Breshears et al. (2011)	+	–	?	+	–	High
Ehlers et al. (2012)	–	–	+	+	–	Medium
Pistohl et al. (2012)	+	–	?	+	–	High
Pistohl et al. (2013)	+	–	?	+	–	High
Beveridge et al. (2017)	–	–	?	+	–	Medium
Taherian et al. (2017)	+	+	+	+	+	High
Norton et al. (2018)	–	–	–	+	–	Low
Jochumsen et al. (2018)	–	–	?	+	–	Medium
Beveridge et al. (2019)	–	–	?	+	–	Medium
Zhang et al. (2019)	–	–	+	–	–	Low
Vařeka (2020)	+	–	?	+	–	High

*+, high-risk of bias; ?, uncertain/non-applicable risk of bias; –, low-risk of bias*.

## Results

### Study Selection and Taxonomy

**Figure 2:**
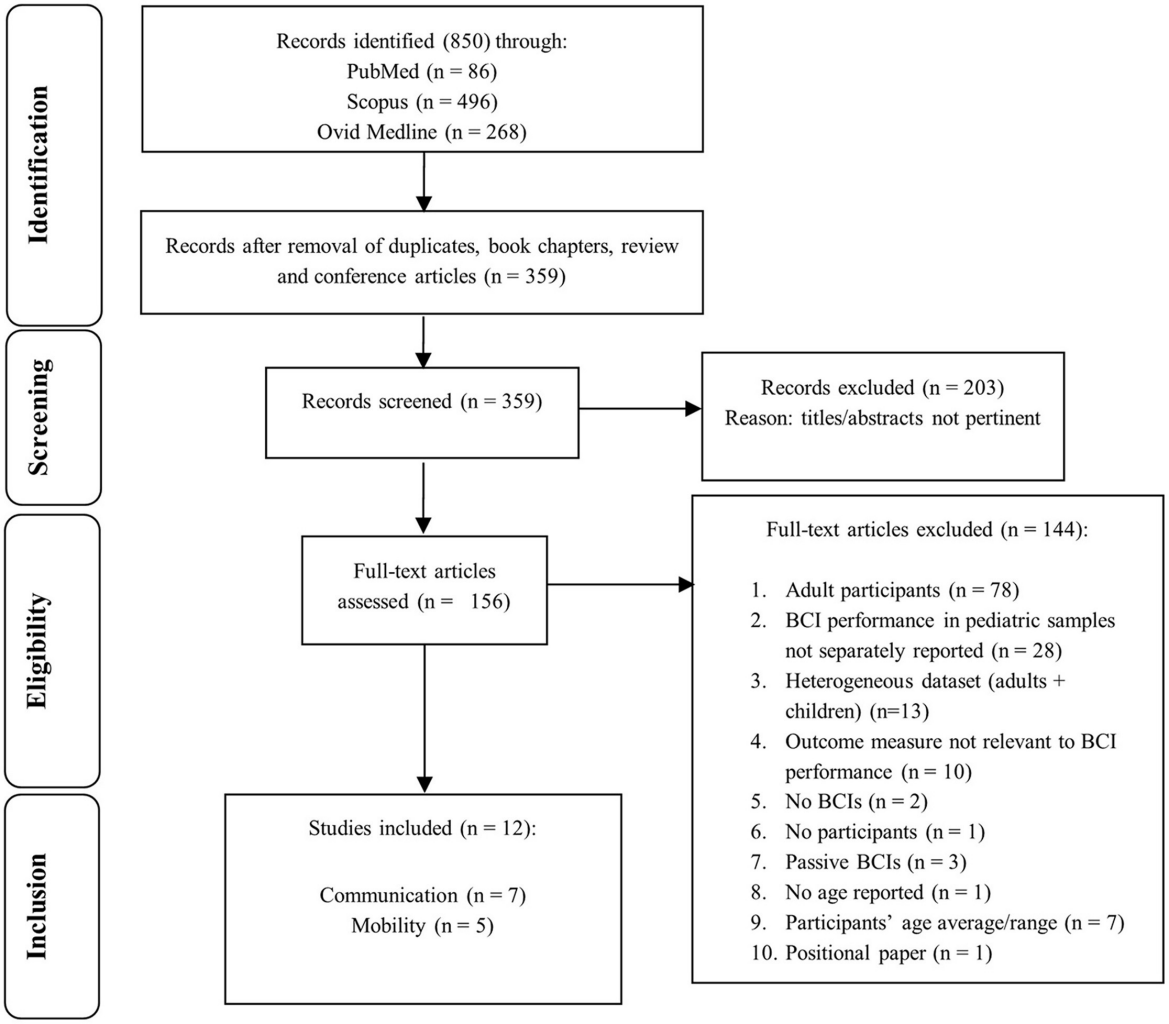
Study selection flowchart. The flow diagram describes identification, screening, eligibility, and inclusion procedures.

The search strategy identified 850 potential papers; 340 duplicates and 151 reviews, book chapters, conference articles were removed. Then 359 titles and abstracts were reviewed and 203 were removed, according to the inclusion criteria (section Screening Process: Inclusion and Exclusion Criteria), leaving 156 articles that required full-text review. Twelve articles were subsequently identified as eligible for inclusion and grouping into sub-categories: seven relating to communication (Beveridge et al., 2017, 2019; Taherian et al., 2017; Norton et al., 2018; Zhang et al., 2019; Vařeka, 2020); and five concerning mobility (Sanchez et al., 2008; Breshears et al., 2011; Pistohl et al., 2012, 2013; Jochumsen et al., 2018). The flowchart in Figure 2 details outcomes of: identification; screening; eligibility; inclusion steps.

**Figure 3:**
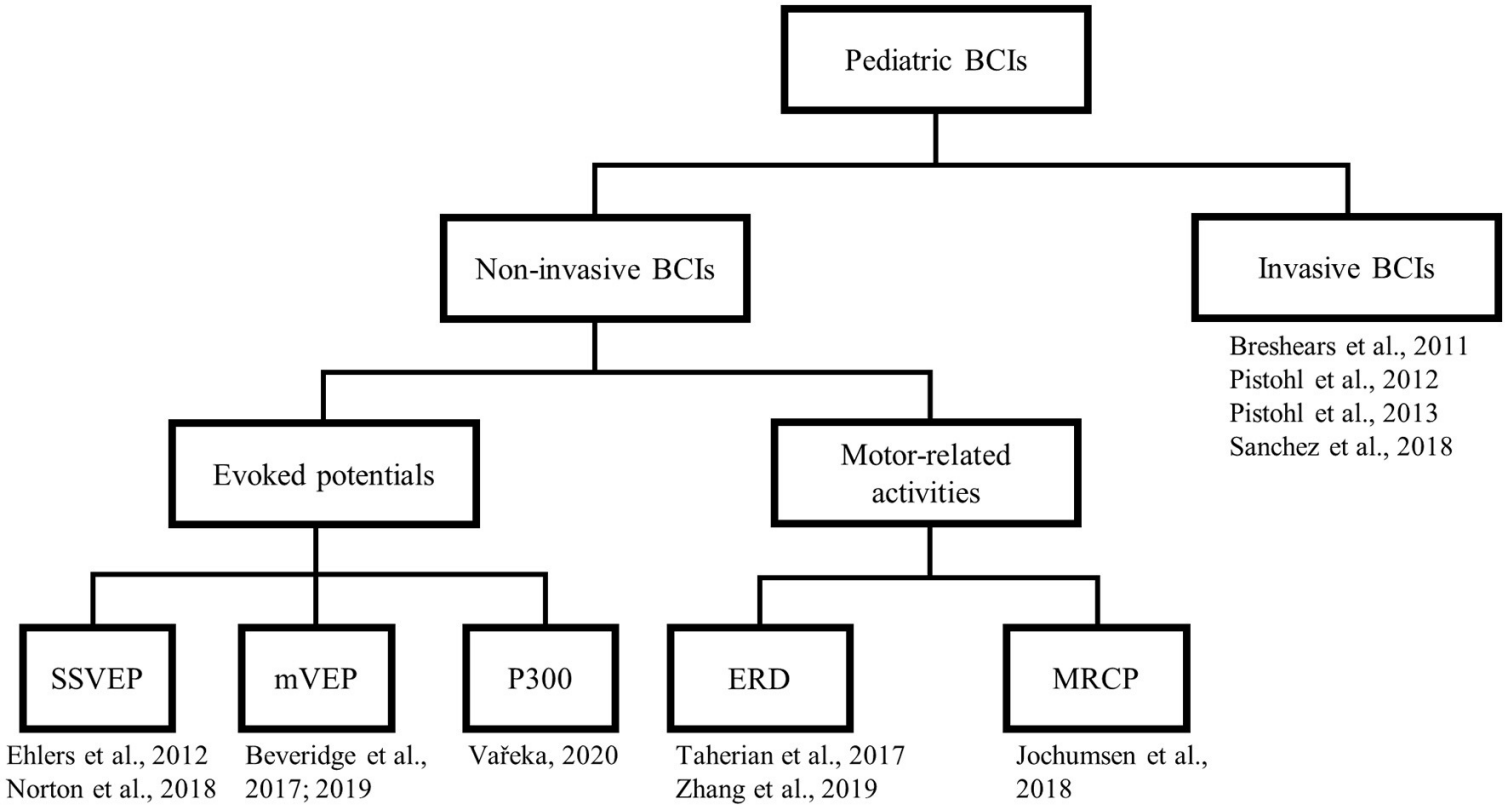
Taxonomy of the selected articles. SSVEP, steady state visual evoked potential; mVEP, motion-onset visual evoked potential; ERD, event-related desynchronization; MRCP, movement-related cortical potential.

We categorized the selected papers (see Figure 3). At the first level of the taxonomy, we grouped papers according to the type of measurement, either non-invasive or invasive. Under the non-invasive category, we further subdivided papers by the type of brain signals harnessed, which includes three types of sensory evoked potentials, MRCP, or event-related desynchronization (ERD). This taxonomy roughly reflects the readiness for clinical translation, with the non-invasive alternatives being more readily implementable. For each study, we adhere to a uniform presentation structure, highlighting the participants, task paradigm, analytical approach, and key findings.

Objectives, participants' information (e.g., age, health conditions, participant number), methods and findings related to online and offline BCI performance are reported in Tables 1–4.

### Non-invasive Pediatric BCIs

Eight studies (Ehlers et al., 2012; Beveridge et al., 2017, 2019; Taherian et al., 2017; Jochumsen et al., 2018; Norton et al., 2018; Zhang et al., 2019; Vařeka, 2020) in this category used EEG as the non-invasive modality for interrogating the pediatric brain. The study by Jochumsen et al. (2018) is the only one on non-invasive pediatric BCI related to manual ability. The other seven non-invasive BCI studies (Ehlers et al., 2012; Beveridge et al., 2017, 2019; Taherian et al., 2017; Norton et al., 2018; Zhang et al., 2019; Vařeka, 2020) focused on new systems to support communication and computer interaction.

#### Evoked Potentials

Five studies harnessed evoked brain responses: steady-state visual evoked potential (SSVEP) (Ehlers et al., 2012; Norton et al., 2018); motion-onset visual evoked potential (mVEP) (Beveridge et al., 2017, 2019); P300 (Vařeka, 2020) following the presentation of a visual stimulus.

Ehlers et al. (2012) investigated the influence of development-specific changes in the background EEG on stimulus-driven BCI with 37 typically developing (TD) children and 14 adults, aged 6–33, using SSVEPs and mouse control and spelling tasks. Only online results but no chance level were reported. Participants navigated a letter matrix to spell six words, two in three different stimuli conditions (low, medium, high frequency), by focusing on one of five target LEDs (corresponding to four directions and a select command) placed around a screen where the letter in the middle of the matrix was highlighted. Participants practiced by spelling their names; however, the youngest participants were assisted by the investigator in locating the target LED given their less developed visual searching abilities. Ehlers et al. (2012) used the Bremen-BCI (Friman et al., 2007) to classify five different SSVEP targets. Poor signals due to insufficient electrode contact were given a low weight or ignored. Classification of signals used to generate the correct-to-complete commands ratio was based on a 2 s sliding window every 125 ms. Accuracies, regarding correct-to-complete commands ratio, were lower than 60% for pediatric participants. Results showed low classification performance (accuracy: ~40%) for the young subjects (age 7–10 years), based on stimulation of 7 and 11 Hz. When a low-frequency (7–11 Hz) visual stimulus was presented to participants, adults consistently achieved higher accuracies (~78%) than those achieved by the three groups of children (group 1 accuracy: ~40%; groups 2 and 3: ~50%). In the medium frequency (13–17 Hz) condition, differences in achieved accuracies were found only between the adults (accuracy: ~78%) and youngest group of children with an average age of 6.73 years (group 1 accuracy: ~55%; group 2: 50%; group 3: 75%). In contrast, no difference between the four groups was found when a high-frequency (30–48 Hz) stimulus was presented (group 1 accuracy: ~38%; group 2: ~45%; group 3: 55%; adults: ~62%). An age-specific shift was observed in the peak synchronization frequency. Peak synchronization increases from 8 to 9 Hz in the lowest age group to 10–11 Hz in adults. Aborted attempts decreased with increasing age and increased as the accuracy level decreased (particularly evident in the high drop-out rates of the youngest age group under low-frequency stimulation). Lastly, the authors discovered the inability to adequately control a BCI using the low-frequency rates. The age factor gains influence with decreasing stimulation frequency.

Similarly, Norton et al. (2018) used the SSVEP paradigm to compare the performance between 15 TD children (aged 9–11) and 11 adults (aged 19–68) in a laboratory environment using a graphical interface. Offline and online results but no chance level were reported. Authors included a minimum offline accuracy requirement for the online analysis. Authors did not specify the number of sessions in their study. We assumed that participants performed only one session preceded by BCI calibration. Participants were asked to focus their attention on three white circles, each alternating between white and black at three different frequencies (6.2, 7.7, and 10 Hz) on the screen. During calibration, participants were directed to focus on the circle highlighted by an on-screen arrow. Participants subsequently repeated the same task without the arrow to test the system online. Norton et al. (2018) applied a calibration phase and a longer experimental phase to classify three different SSVEP targets. If the calibration phase accuracy was <85%, the participant could not proceed to the experimental phase (online phase). Eleven children and all adults met the minimum accuracy requirement. Children and adults achieved similar performance during the experimental phase (accuracy: 79 vs. 78%; latency: 2.1 vs. 1.9 s; bitrate: 0.05 vs. 0.56 bits s^−1^). Feature extraction and classification were based on the canonical correlation analysis (CCA) and used to determine the SSVEP targets. Norton et al. (2018) used a method similar to that applied by Lin et al. (2006) wherein EEG signals from multiple channels were used to calculate the CCA coefficients considering the stimulus frequencies in the systems. The frequency with the highest coefficient indicates the SSVEP frequency. This study demonstrated that children can use an SSVEP-based BCI with higher accuracy (average accuracy: 79%) than Ehlers et al. (2012) when low-frequency stimulus is applied. However, their good performance could be dependent on the different environments. Participants completed a target selection task and not a text-entry task. Also, they applied different stimulus frequencies.

Beveridge et al. (2017) evaluated whether 15 TD adolescents (aged 13–16) could gain control of an mVEP-BCI paradigm for video game playing. Offline and online accuracy of target vs. non-target stimuli classification (chance level: 50%) and 5-class discrimination results (chance level: 20%) were reported. Participants were engaged in a 3D car racing video game that involved changing lanes at several checkpoints performing three laps. As participants approached a checkpoint, one of the five motion stimuli was presented above each of five lanes with an arrow indicating the target lane for positioning the car. Participants attended to the motion stimulus associated with the target lane. If the target lane was selected correctly by the BCI, participants were rewarded with points and speed boost. The authors collected 300 mVEP trials from 12 gel-based EEG electrodes to calibrate a classifier tested with additional 300 trials. Beveridge et al. (2019) subsequently reported performance achieved by BCI-naïve and BCI-experienced adults with a near-identical protocol. For this review, Beveridge et al. (2017) and Beveridge et al. (2019) were considered identical as they relied on the same adolescent dataset. The two studies by Beveridge et al. (2017, 2019) report results from a single adolescent mVEP dataset. The collected mVEP data were resampled from 250 to 20 Hz and filtered. Data were averaged over five trials to generate 12 feature vectors for each stimulus which corresponded to the 12 EEG channels. Offline and online accuracies and information transfer rate (ITR) were reported for each participant. Participants achieved 85.17% offline accuracy through a leave-one-out cross-validation, and 68% accuracy and 11 bits per minute during online trials. The authors reported Cz, P7, and O1 as the most discriminative channels across participants. Beveridge et al. (2019) also compared the group of BCI naïve teenagers with nine BCI naive adults. Adults achieved higher classification accuracies compared to teenagers (average accuracies in 3 laps: 75.4 vs. 68%), but the difference between adults and teenager was significant only in the third lap.

Lastly, Vařeka (2020) entailed large-scale offline analysis of P300 visually-evoked potential signals collected from 250 children (aged 7–17) without any identifying physical symptoms, playing “Guess the Number.” This game requires participants to focus on a self-selected number between 1 and 9 as a series of numbers (1–9) flash on a screen in random order. When flashed, the selected number elicits a P300 response. Thus, the algorithm predicts the selected number. Offline results but no chance levels were reported. Vařeka (2020) collected 532 trials using three channels. The study aimed to compare convolutional neural networks (CNNs) against linear discriminant analysis (LDA) and support vector machines (SVM). The author applied a baseline to correct each epoch and eliminated epochs containing amplitudes exceeding 100 μV. Epochs were divided into 20 equal-sized intervals wherein the amplitudes were averaged. Features were classified separately using CNN, LDA, and SVM. All classifiers produced similar classification accuracies. Single-trial classification accuracies ranged between 62 and 64%, while trial averaging raised accuracies to 76–79%. Precision, recall, and area under a receiver operating characteristic (ROC) curve (AUC) were 61.5–63.5%, 60.5–67.5%, and 62–66%, respectively, for single-trial classification. Averaging groups of one to six neighboring epochs instead of single trials improved classification accuracies.

#### Motor-Related Activities

Three studies investigated motor-related activities with children (Taherian et al., 2017; Jochumsen et al., 2018; Zhang et al., 2019).

Two studies applied a MI paradigm (Taherian et al., 2017; Zhang et al., 2019) and EMOTIV system, a commercially available headset. Although both studies reported the use of the same EEG device (EMOTIV Epoc®), Zhang et al. (2019) referred to a dry system while Taherian et al. (2017) described a wet device. Zhang et al. (2019) reported that the electrode foam pads were immersed in a saline solution to ensure reliable connection before being placed on the child's head. Both studies should have described the EMOTIV Epoc® as a headset with saline-soaked felt pads. These sensors are not wet in the traditional sense, but they are not considered truly dry. Both studies explored the possibility of implementing an EEG-based visual motion BCI and they used the Emotiv Software Development Kit (SDK) for the analysis and classification. Both studies extracted modulation features. Classification results were obtained based on ERD phenomenon.

Taherian et al. (2017) evaluated the feasibility of implementing an EEG-based BCI using the 14-saline-based electrode version of this headset in five children (aged 9–17) with spastic quadriplegic CP. The EMOTIV is packaged with software that provides visual feedback of cognitive tasks and a gamified training protocol. Participants donned the EMOTIV Epoc® headset and completed six 30-min training sessions where they were guided through the EMOTIV software to move a virtual cube up, down, left, or right using MI of the limbs. EEG signals were processed using Cognitiv™ Suite provided by EMOTIV Epoc®. The Cognitiv system processes the brainwaves and matches them to the patterns of thought trained, relying on ERD, detected on the EEG signals within the frequency range of 0.2 and 43 Hz (Lang, 2012). The authors developed a puzzle game and participants were asked to complete the puzzle after each training session. The puzzle was completed in an online paradigm using the same MI tasks from the training sessions while continuous visual feedback was provided. When participants were able to produce MI tasks with precision, they were rewarded with a puzzle piece. Participants completed five to seven sessions. Unfortunately, Taherian et al. (2017) reported only online performance scores for left and right arm for some participants in graphs and do not report accuracy, latency, or bitrate. Performance values were approximated and based on graph readings. All participants experienced challenges following protocol for various reasons related to their condition, including: difficulties focusing; seizures during trials; anxiety; equipment discomfort; lack of enjoyment when playing. All pediatric participants demonstrated inconsistent and unreliable control of BCI. They concluded that existing commercial BCIs are not designed according to the needs of end-users with CP.

Zhang et al. (2019) conducted a cross-over interventional study on 26 TD children (aged 6–18) to estimate the performance of two tasks (driving a remote-control car and moving a computer cursor). Children participated in two sessions where they performed a MI task (imagery of opening and closing both hands to move the car or the cursor) and a “goal-oriented thought” task (think of moving the car or the cursor toward a target). During each session, participants completed eight trials as training data for the BCI followed by 10 testing trials to evaluate the system performance. The BCI's goal was to complete the designated task (car or cursor) using one of two strategies (MI or goal-oriented thought). We assumed that Zhang et al. (2019) used the Emotiv SDK to extract ERD features using an 8-s window. They reported good performance using the EPOC headset and Radial Basis Probabilistic Neural Network to distinguish between baseline and training epochs. Zhang et al. (2019) reported online results (chance level: 70% classification accuracy, 0.4 Cohen's kappa) in terms of Cohen's kappa scores (range 0.025–0.90). Performance correlated with increasing age, but sex was not associated. A Cohen's kappa of 0.4 or higher indicated successful control.

Jochumsen et al. (2018) deployed motor execution tasks to elicit MRCP in the motor cortex. MRCP is an event-related potential locked to the onset of a movement, reflecting the preparatory brain activity. They detected MRCPs in 8 adolescents with hemiplegia or diplegia CP (aged 11–17) *via* EEG, with the goal of maximizing motor learning by temporally aligning afferent feedback with the cortical manifestation of movement intention. Offline classification accuracy (chance level: 60–65%) was reported. Participants dorsiflexed an ankle at self-determined pace during a single 15-min session. Electromyographic signal was used to divide continuous EEG into epochs. Mean amplitudes, absolute band power, and template matching were extracted from each channel after filtering. Template matching was obtained by calculating the cross-correlation between the template of the movement epochs (averaged epochs for each channel for each participant) and the epochs. A random forest discriminated movement intention epochs from idle epochs using a leave-one-out cross-validation and achieved up to 85% accuracy.

For study objective, population, and tasks of non-invasive BCIs see Table 1. For signal processing techniques and results see Table 2.

### Invasive Pediatric BCIs

Four articles (Sanchez et al., 2008; Breshears et al., 2011; Pistohl et al., 2012, 2013) included in this category used ECoG as invasive modality for interrogating the pediatric brain. Three of these studies applied motor execution paradigms while one additionally invoked MI (Breshears et al., 2011). Studies recruited individuals with epilepsy who had implanted electrode grids used to monitor brain activity prior to surgery.

First invasive pediatric BCI study was presented by Sanchez et al. (2008). They explored motor control paradigm with two adolescents aged 14 and 15 years who had an electrode array implanted to monitor their intractable epilepsy prior to surgery. Participants engaged in six repetitions of arm reaching and pointing tasks. ECoG signals were decoded from pre-motor, motor, somatosensory, and parietal cortices using a linear adaptive finite impulse response (FIR) filter trained with Wiener solution. Sanchez et al. (2008) reported the first example of the ability to decode pediatric ECoG signals for an online BCI model. They processed ECoG signals collected during reaching and pointing task by first filtering the data between 1 and 6 kHz. Features from each channel were fed into the above FIR filter topology to generate estimate of arm trajectory. Pearson's correlation was used to determine how closely the decoded signals matched the true arm's trajectory. The highest Pearson's correlations were achieved using the 300 Hz−6 kHz frequency band feature.

Breshears et al. (2011) demonstrated the decodable nature of ECoG signals from motor and/or language (Wernicke's or Broca's area) cortices by six pediatric participants (aged 9–15) who required invasive monitoring for intractable epilepsy. To move a cursor on the screen, children performed a motor (e.g., hand opening and closing, repetitive tongue protrusion) or phoneme articulation (oo, ah, eh, ee) task. Participants were asked to move the cursor along one dimension to hit a target on the opposite side of the screen during a single online session. Trials were grouped into runs of up to 3 min with a rest period of <1 min. Breshears et al. (2011) applied an autoregressive spectral estimation in 2 Hz bins ranging between 0 and 250 Hz to decode ECoG signals. For each electrode and frequency bin, power increases or decreases in the significant task-related spectral power were identified by calculating the r^2^ correlation between baseline spectra and activity spectra for each active task. Online performance accuracy (chance level: 50%) was calculated considering the number of successes (i.e., hit the target) divided by the total number of movements after each block. Results were compared to a previous study (Leuthardt et al., 2004; Wisneski et al., 2008) conducted with five adult participants (aged 23–46). The results showed that the pediatric participants' performance matched the adults' one and signals can be decoded and affected in the same way as adult brain signals. Within 9 min of training, children achieved 70–99% target accuracy in experiments where multiple cognitive modalities were used to achieve an imagined action to control a cursor on the screen. Children controlled the cursor using hand movements using β (15–40 Hz) and γ (60–130 Hz) frequency ranges and, two with tongue movements using high-γ (107.5–155 Hz) frequency range. Four of the six participants began with achieved accuracies <70%. Two participants were able to generate BCI control using imagined movement rather than over performance of the task. The mean accuracy was 81% and the mean training time was 11.6 min. The adult group required 12.5 min and reported a mean accuracy of 72%. Table 4 outlines the range of accuracies for each participant using one or different movements. These findings form proof of concept that decoding signals from the pediatric cortex is possible and may be used for BCI control.

Turning attention specifically to grasping movements, Pistohl et al. (2012) conducted a single-session of study with three pediatric participants (aged 14–16) who had electrodes implanted for pre-surgical epilepsy diagnostics. Participants' self-initiated reach-to-grasp of a cup with either precision or whole-hand grip, relocated the cup and finally returned their hand to a central resting position. Participants completed between 303 and 338 trials. Pistohl et al. (2012) focused on two-class classification of precision grip and whole-hand grip on offline analysis (chance level: 50% classification accuracy). Common average reference and low-pass filtering were applied. The authors utilized an rLDA classifier and reported decoding accuracy and temporal evolution of grasp discriminability. The three participants achieved between 84 and 97% decoding accuracy. Temporal evolution of grasp discriminability was 0.2 s.

Subsequently, Pistohl et al. (2013) utilized the same data to automatically detect the time of grasping movements within a continuous ECoG recording. After filtering, authors extracted frequency band amplitudes and trained an rLDA classifier to distinguish two classes, occurrence of the grasp and no grasp. Ten-fold cross-validation was used to test detection performance for each subject. Offline results were reported. Based on the previous work, we assumed that the chance level considered was 50% but the authors did not report it. Results showed amplitudes recorded in the high-gamma range from hand-arm motor-related channels were used to achieve the best performance. Local maxima between 56–128 Hz and 16–28 Hz. Low-pass filtered components, 16–28 and 56–128 Hz amplitudes reported best classification results when used together to feed the classifier. Sensitivity and specificity depended on temporal precision of detection and on the delay between event detection and when the event occurred.

For study objective, population, and tasks of invasive BCIs see Table 3. For signal processing techniques and results see Table 4.

### Quality Assessment and Risk of Bias

**Figure 4:**
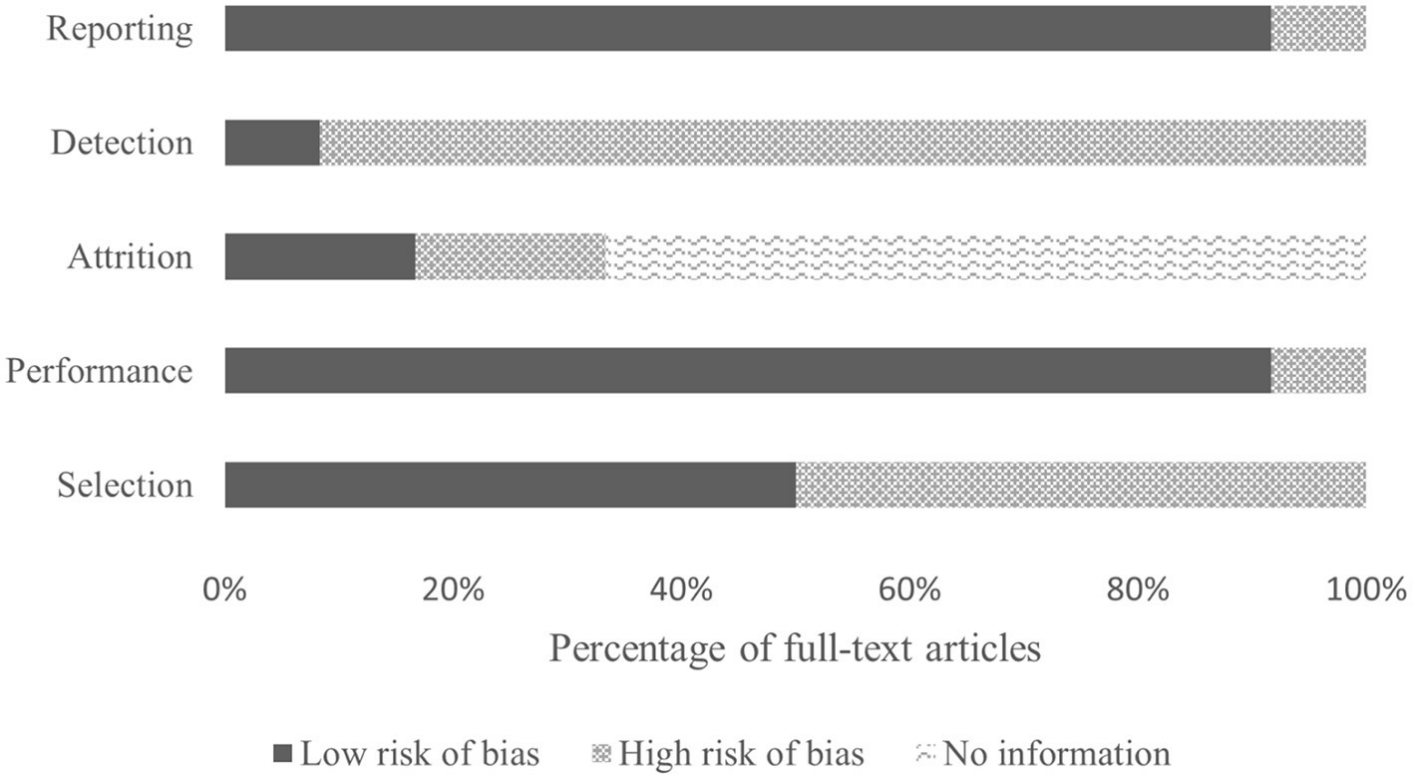
Bar plot visualization of risk-of-bias assessments.

The quality of the included studies ranged from 45% (Taherian et al., 2017) to 91% (Zhang et al., 2019), with a median score of 0.70 and an interquartile range of 0.60–0.76. For breakdown of quality, appraisal markings see Table 5. Ten included papers present primary exploratory research using a multiple case study design, and two present cross-sectional studies (Ehlers et al., 2012; Zhang et al., 2019). Overall, the quality of the studies was adequate. One study was assessed as limited (Taherian et al., 2017), six as adequate (Sanchez et al., 2008; Breshears et al., 2011; Pistohl et al., 2013; Beveridge et al., 2017, 2019; Norton et al., 2018), three as good (Ehlers et al., 2012; Pistohl et al., 2012; Jochumsen et al., 2018), and two as strong (Zhang et al., 2019; Vařeka, 2020). However, some of the algorithms used by Zhang et al. (2019) are proprietary, making it difficult for the reproducibility of the experiments because the EMOTIV SDK may require buying a license to access some of the APIs. For the quality assessment of the signal processing, we considered sufficient reporting information about the features extracted and the classification algorithms applied. Also, we did not take into account open science (data and software/code sharing) and the reproducibility of the obtained results for the quality assessment. We highlight that Vařeka (2020) is the only included study that made publicly available data and software code. Table 6 and Figure 4 report the domain-level judgments for each study and a summary bar plot of the distribution of the risk-of-bias assessment within each bias domain. Breshears et al. (2011), Ehlers et al. (2012), Norton et al. (2018), and Beveridge et al. (2019) are the only studies comparing children to adults. Only six studies (Ehlers et al., 2012; Beveridge et al., 2017, 2019; Jochumsen et al., 2018; Norton et al., 2018; Zhang et al., 2019) considered important inclusion and exclusion criteria for selection bias: information related to the dominant hand; previous experience with BCI; use of medication; individual participants' age; gender; history of brain injury. Four studies (Sanchez et al., 2008; Breshears et al., 2011; Pistohl et al., 2012, 2013) included children with epilepsy but did not control for epilepsy-related brain activity or differences in the electrode positioning. Vařeka (2020) required large numbers of participants but did not report individual participants' ages or handedness or report results for male vs. female. In terms of maintaining fidelity to the study protocol, nine studies implement the same protocol consistently across participants. Three studies applied protocols including a different number of sessions and trials across participants (Breshears et al., 2011; Ehlers et al., 2012; Taherian et al., 2017). Missing data (e.g., participants who dropped out or researchers' excluded trials or low performance) were considered and handled appropriately only in one study (Norton et al., 2018). Three studies took missing data into consideration but did not discuss or analyze them appropriately (Ehlers et al., 2012; Taherian et al., 2017; Zhang et al., 2019). We did not consider questions related to assessors blinded to the intervention or exposure status of participants, because blinding is not appropriate for BCI studies. Two studies did not assess outcomes using valid and reliable measures (Ehlers et al., 2012; Taherian et al., 2017). Ehlers et al. (2012) reported an unclear definition of the performance evaluation (e.g., correct-to-complete commands ratio). Taherian et al. (2017) did not include a reliable measure in their protocol (i.e., performance score). Table 7 summarizes the performance evaluation metrics used in the 12 studies in this review. In terms of confounding variables assessed, we considered participants': age; gender; fatigue; psychological factors. Only one study did not introduce bias through confounding variables (Zhang et al., 2019). One article did not pre-specify outcomes (Taherian et al., 2017). Regarding bias that might affect these 12 studies, we highlight only five studies reported concerns due to the small sample sizes (Sanchez et al., 2008; Breshears et al., 2011; Jochumsen et al., 2018; Zhang et al., 2019). Vařeka (2020) is the only study that recruited many participants. Self-reporting risk of bias was applicable only for one study (Zhang et al., 2019), which included: questionnaires for psychological and cognitive information; BCI workload.

**Table 7:** Performance evaluation metrics used in the 12 studies on pediatric BCIs.

**References**	**Performance**
Sanchez et al. (2008)	→Accuracy
Breshears et al. (2011)	→Accuracy
Ehlers et al. (2012)	→Accuracy
Pistohl et al. (2012)	→Correlation coefficients
Pistohl et al. (2013)	→TPR/FPR/FP-rate
Beveridge et al. (2017)	→Accuracy → ITR
Taherian et al. (2017)	→Performance score
Jochumsen et al. (2018)	→Accuracy
Norton et al. (2018)	→Accuracy → Latency → Bitrate
Beveridge et al. (2019)	→Accuracy → ITR → Latency
Zhang et al. (2019)	→Cohen's kappa
Vařeka (2020)	→Accuracy → Precision → Recall → AUC

*FPR, false positive ratio; TPR, true positive ratio; FP-rate, false positive rate; AUC, area under ROC curve; ITR, information transfer rate*.

### Evaluation Factors

Evaluation factors usually reported for BCI studies are: usability; performance; user's satisfaction; evaluation of psychological factors; brain workload; fatigue; quality of life; cognitive evaluation (Nicolas-Alonso and Gomez-Gil, 2012; Choi et al., 2017). Only one study reported subject fatigue using a 5-point Likert scale questionnaire (Zhang et al., 2019). Zhang et al. (2019) used a self-report questionnaire to investigate the psychological factors of participants during BCI experiments. Taherian et al. (2017) justified the absence of self-report questionnaires due to the severity of participants' conditions and limited communication capabilities. In terms of performance, most of the studies reported accuracy rates (see Table 7). Norton et al. (2018) was the only study with a self-report questionnaire for the usability of the BCI system.

## Discussion

### Pediatric BCIs

The primary objective of this systematic review was to examine studies related to the use of BCIs in pediatric populations. We described the current state-of-the-art for pediatric BCIs and assessed the quality and the risk of bias of the 12 articles. The included studies raise several challenges addressed in the following sections where we describe considerations for future research to make BCI technologies suitable for children. We also identify requirements to render BCIs suitable for clinical translation.

#### BCIs for Communication: State-of-the-Art

Regarding studies investigating BCIs for communication, a wide range of methods were implemented yielding various levels of success. Seven studies involved non-invasive EEG as the signal acquisition modality (Ehlers et al., 2012; Beveridge et al., 2017, 2019; Taherian et al., 2017; Norton et al., 2018; Zhang et al., 2019; Vařeka, 2020). Five studies analyzed evoked potentials (Ehlers et al., 2012; Beveridge et al., 2017, 2019; Norton et al., 2018; Vařeka, 2020) and two used movement-related potentials as the control signal (Taherian et al., 2017; Zhang et al., 2019).

Two studies took advantage of the active mental task MI (Taherian et al., 2017; Zhang et al., 2019). Taherian et al. (2017) deployed a consumer-grade EEG headset with five youth with spastic quadriplegic CP to decode left and right arm MI. This was the first study that involved children and computer access with a commercial EEG-BCI using the EMOTIV Epoc® hardware, but participants achieved poor accuracies (0.08–0.56 peak performance score range). Zhang et al. (2019) utilized the same low-cost commercial EEG headset as Taherian et al. (2017) to compare MI and “goal-oriented thinking” as tasks to control either a toy car or computer cursor with TD children. Participants achieved an online Cohen's kappa score of 0.46 pointing to successful control of the BCI. Importantly, they found performances were higher when users were controlling toy car vs. computer cursor. These results were attributed to the increased engagement of the children when controlling the car. This study points to the potential of low-cost BCIs being successfully used as a binary switch with pediatric users. It is unclear whether the poor results achieved by Taherian et al. (2017) are due to neurological differences of children with CP compared to TD children. It is possible that the physical and cognitive limitations, common among children with CP, were the source of differences in achieved accuracies.

Many limitations in current methods emerge when translating BCIs for communication to the target population (e.g., children with severe disabilities). For example, in the study by Taherian et al. (2017), participating children had unique head shapes that limited the ability of the electrodes on the BCI to gain contact with the scalp. Additionally, individuals with CP have been known to produce large muscular artifacts due to involuntary movements. Since the authors were unable to record raw EEG data, it is unclear whether artifacts disrupted signal acquisition, ultimately affecting their training data. The embedded EMOTIV system used by Taherian et al. (2017) may not have adequate artifact reduction methods, which would significantly affect the classification of the signals. Lastly, the authors reported another issue due to the severity of participants' conditions. They found many difficulties conducting 30 min training sessions and mentioned the impossibility of collecting enough EEG data to adequately train the classifiers. Moreover, participants were unable to learn to reproduce specific MI tasks within the timeframe of the study. In contrast, Zhang et al. (2019) demonstrated that a goal-oriented strategy works better than MI task with children and it may be useful for teaching MI tasks to children with disabilities. They found a BCI illiteracy rate higher in children than in adults and emphasized the potential difficulty children experience when reproducing their thought strategy in each trial. These issues might be resolved by including additional training phases in the study acquisition protocol for pediatric BCIs. The lack of customization of commercial headsets for pediatric head sizes may also justify reduced performances of children as compared to adults. For this reason, Zhang et al. (2019) had many difficulties placing the electrodes in locations dictated by the international 10–20 system in pediatric BCI studies.

The other five communication-focused studies utilized the reactive tasks known as SSVEP (Ehlers et al., 2012; Norton et al., 2018), mVEP (Beveridge et al., 2017, 2019), and P300 (Vařeka, 2020). The two studies investigating SSVEP (i.e., where the user visually fixates on a flashing target to indicate its selection) utilized the EEG signal acquisition modality and achieved mixed results. There are three main performance measurements of an SSVEP-based BCI: accuracy (probability of predicted target matching the target), latency (mean time from target onset to classification), and bit rate (transfer information rate, e.g., the amount of information conveyed per time unit). Ehlers et al. (2012) tested an SSVEP-BCI with five visual targets achieving quite poor results with 40 TD children. Accuracies ranged from 38 to 75%. Results reported by Ehlers et al. (2012) demonstrated that mean accuracy rates depend on age and frequency of the stimulation (10–11 Hz). The adult comparison group obtained consistently higher accuracy rates compared to all three children samples and an age-specific shift can be seen in the peak synchronization frequency. Their findings align with those reported by Roland et al. (2011). Using an ECoG and EEG-BCI, Roland et al. (2011) found that higher frequency bands show significant correlations with age in participants aged 11–59 years. This full-text was excluded because authors did not report BCI performance. Norton et al. (2018) built upon the work of Ehlers et al. (2012) by improving an SSVEP-BCI for TD children. Eleven of the 15 children exceeded the threshold of successful BCI control during offline sessions and attained an average of 79% online classification accuracy. This result was statistically similar to results achieved by the participating adult cohort. While the achieved bit rates of pediatric participants were lower than adults, this study points to the promise of successful control of SSVEP-BCIs by pediatric users. As noted in Norton et al. (2018), there are many methodological differences between their study and Ehlers et al. (2012), which may explain result differences. Methodological discrepancies include differences in the task controlled by the BCI, slightly different stimulation frequencies, involvement of a calibration phase, dissimilar environmental settings, and exclusion of participants after a poor calibration phase. Lastly, Norton et al. (2018) is the only study where performance is reported in terms of accuracy, latency, and bit rates. The latency is the average amount of time between the onset of the stimuli and the classification of the predicted target (Norton et al., 2018). Norton et al. (2018) showed that children were slower than adults, although this result was not significant.

The two studies by Beveridge's group (Beveridge et al., 2017, 2019) involve racetrack video games and are the first studies with pediatric subjects using mVEP-BCI applications. The overarching goal of these two articles is to explore a BCI task that is less visually fatiguing than commonly investigated alternative BCI tasks such as SSVEP and P300. They demonstrated the feasibility of mVEP paradigm achieving an average accuracy of up to 72% but did not apply any measurement or questionnaires to evaluate visual fatigue among participants. The absence of a qualitative and quantitative evaluation of visual fatigue limits the reliability of these two papers despite the high performance reported.

Vařeka (2020) is the first study that includes a large group of pediatric participants (e.g., 250) and that applied deep learning algorithms (e.g., CNN) in BCIs for children. The study showed that LDA, SVM, and CNN had similar classification performance (62–64% accuracy) using P300 features. The article reports higher performance (~77% accuracy) when the BCI employs averaging of P300 trials. Comparing trial groupings of various sizes (1–6 trials), average classification accuracy increases with each group size increase. Vařeka (2020) reports an important limitation of CNN for BCIs: LDA and SVM showed faster computational time than CNN (300 ms, 1,600 ms vs. 46 s CPU/26 s GPU). Vařeka (2020) is the only study conducted in a school setting. The authors suggest that the school setting likely hindered the children's performance. This is corroborated by the fact that 30.3% of epochs were rejected due to noise. Artifact correction was not possible due to the limited number of EEG channels used (only three electrodes). The authors could have employed a larger number of channels to increase spatial resolution and likely also the performance accuracy.

#### BCIs for Mobility: State-of-the-Art

Five studies exploring BCIs for mobility have involved children in the last 12 years (Sanchez et al., 2008; Breshears et al., 2011; Pistohl et al., 2012, 2013; Jochumsen et al., 2018). Four of the five studies utilized invasive techniques and acquired ECoG signals (Sanchez et al., 2008; Breshears et al., 2011; Pistohl et al., 2012, 2013).

Sanchez et al. (2008) investigated ECoG amplitude modulation for motor control tasks (e.g., reaching and grasping) for the first time with a small group of pediatric participants. Sanchez et al. (2008) generated online predictive models to decode motor commands in the primary motor cortex. The predicted trajectories showed moderate correlations with actual trajectories. However, the estimates involved very large variances representing the models' inability to distinguish the motor activity from noise in a realistic setting.

Breshears et al. (2011) conducted the first study on pediatric ECoG-BCI presenting a comparison of results between adult and pediatric participants. Breshears et al. (2011) demonstrated that recent advances in neuroprosthetic research may be applied to BCI applications with children. The technology is ready to move beyond single case studies to be tested in wide-spectrum experimentation of BCI for mobility involving a pediatric population. They showed that prepubescent and peripubescent children can rapidly and effectively achieve control of a computer cursor (accuracy: 70–99%), after short training times of 8–18 min, using a multitude of different cognitive modalities and their associated cortical physiologies. Although neurofeedback on brain signals has been used previously with children, its use was primarily for diagnostic and therapeutic purposes, rather than the express purpose of control alone.

Pistohl et al. (2012, 2013) reported good performance in arm and grasp movement prediction with their ECoG-BCI. The authors recorded from hand and arm areas of the human motor cortex as sites likely to be utilized in future BCI applications. While predictions were quite reliable at sub-second precision, the observed temporal deviations might still be too large for applications requiring very precisely timed movement control. Their grasping detection method based on linear discriminant analysis on ECoG recordings from the motor cortex can predict events 125–250 ms before their occurrence, without accuracy loss.

Jochumsen et al. (2018) investigated motor preparation and execution tasks performed by children with CP and collected with EEG. This is the only non-invasive mobility study captured by this review. Participants achieved accuracies as high as 85% when classifying ankle dorsiflexion activity vs. idle. Jochumsen's group also demonstrated that children with CP can generate motor-related cortical potentials that are visually discernable, creating the possibility of motor decoding using non-invasive modalities such as EEG with children with CP.

### Pediatric Participants and Sample Size

The 12 publications included were specifically targeting BCIs for use with children (Sanchez et al., 2008; Breshears et al., 2011; Ehlers et al., 2012; Pistohl et al., 2012, 2013; Beveridge et al., 2017, 2019; Taherian et al., 2017; Jochumsen et al., 2018; Norton et al., 2018; Zhang et al., 2019; Vařeka, 2020). These studies generally included a limited number of pediatric participants and combined children and youth. Three of the papers compared adults and children (Ehlers et al., 2012; Taherian et al., 2017; Beveridge et al., 2019), however only Beveridge et al. (2019) discussed possible differences in performance due to cognitive and neurological differences between participants. Thirteen full-text articles were excluded because they included only a few pediatric participants among a heterogeneous age group, without developing a specific protocol for pediatric ages (e.g., Pistohl et al., 2008; Schalk et al., 2008; Perego et al., 2011; Milekovic et al., 2012; Zhang et al., 2013). An additional 28 papers were excluded due to the inclusion of a homogenous group of adults and children, without special consideration for differences between the two groups of participants (e.g., Treder et al., 2011; Weyand et al., 2015). It is critical that researchers acknowledge the physiological differences between adults and children when studies involve participants spanning the pediatric and adult age ranges.

**Figure 5:**
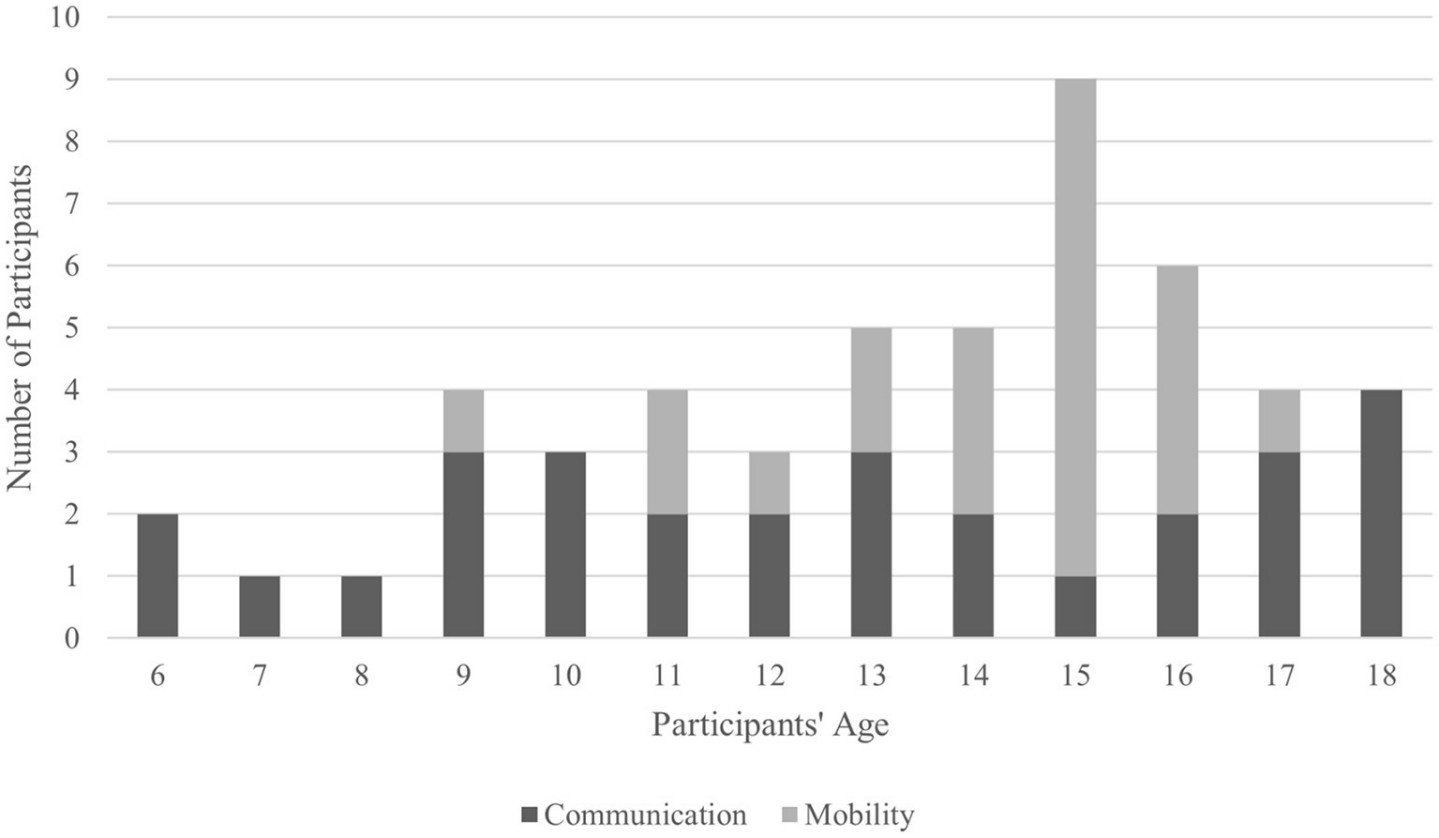
Age distribution in pediatric BCIs. Ehlers et al. (2012), Norton et al. (2018), Beveridge et al. (2017, 2019), and Vařeka (2020) were not considered as they did not provide a specific age breakdown for their participants.

Overall, the sample sizes of pediatric participants in the studies were small, ranging from two (Sanchez et al., 2008) to 250 (Vařeka, 2020). Half of the studies involved fewer than 10 pediatric participants (Tables 1, 3). Only one study exceeded 51 participants (Vařeka, 2020). Despite a large sample size, Vařeka (2020) did not perform any statistical analysis among results nor did they stratify participants into small age groups to determine effect of age on performance. Median number of pediatric participants in reviewed studies was 11. In total, 370 pediatric participants were enrolled across 12 studies, with 250 contributed by Vařeka (2020). Excluding Norton et al. (2018) who did not report participant sex, 42% of children and youth were female. Average age of pediatric participants across studies was 13.3 ± 3.2 years. For mobility-related BCI studies, most of the participants clustered around mid-adolescence (14–16 years) while for communication BCI studies, participants were mostly scattered between 9 and 18 years. Notably, across all studies, only four participants were <9 years old. Figure 5 shows the age distribution of pediatric participants excluding Ehlers et al. (2012), Beveridge et al. (2017, 2019), Norton et al. (2018), and Vařeka (2020). Age data extracted from Zhang et al. (2019) data were interpolated and extracted from a graph reported in the article. Two studies (Ehlers et al., 2012; Norton et al., 2018) reported only the average of participants' ages. Future studies should continue to increase sample sizes, as this will allow for more powerful investigations of age on performance.

#### Inclusion of Individuals With Disabilities

It is important to note the scarcity of studies involving individuals with disabilities in both the pediatric and adult BCI literature. Kübler et al. (2013) stated that <10% of published BCI papers include participants with severe motor disability, despite this group being the ultimate target population of the research. Among the papers examined in this review, only Taherian et al. (2017) and Jochumsen et al. (2018) included participants of the target population (namely, CP) of pediatric age. Jochumsen et al. (2018) involved youth with CP performing a motor task and found that the participants demonstrated MRCP. Researchers questioned whether MRCP could be discriminated due to the atypical movements and reorganized motor cortical networks of individuals with CP (Papadelis et al., 2018).

The progression of research involving adult participants has progressed from involving typically developed adults to including adults with disabilities. Just as it has been for BCI studies involving adult participants, BCI research projects should first include able-bodied children and then immediately extend to children with disabilities. BCI studies should be designed as prospective cohort studies with strong experimental designs involving pediatric participants with severe motor disabilities rather than solely TD controls. Several studies have investigated the use of BCIs for attention-deficit hyperactivity disorder treatment in children showing promising results (Kulseng et al., 2007; Sigurdardottir et al., 2010; Felton et al., 2012; Rohani et al., 2014; Gabis et al., 2015). P300 is typically used in this training and the successful results indicate that the BCIs can accurately distinguish P300 signals in the participating children. Unfortunately, we could not include these studies because they were published in conference proceedings or they did not report BCI performance measures.

#### BCI User Experience of Children

So far, studies have only reported that photosensitivity of some children may preclude the use of some BCI paradigms. None of the studies in this review reported any adverse events. Potential disadvantages of BCIs include the time and effort required to learn to use a BCI system and the speed at which information can be transferred. Additionally, the inconvenience of the setup and cleanup of the hardware associated with the technology as well as its discomfort and portability may compromise integration into daily life. These considerations will need to be balanced with the promise that BCI holds to facilitate communication and quality of life for people who have explored all other options available. Researchers have the responsibility to provide accurate and balanced information for the young potential participants and their families. Assent should be encouraged, and long-term follow-up embedded within the study design. Moreover, as reported in a notable review on augmentative and alternative communication (AAC) by Akcakaya et al. (2014, p. 24): “some children with disabilities would certainly benefit from using BCIs for communication and control, and researchers should begin to investigate this possibility.” BCIs have the potential to be used as assistive technology devices for pediatric users but only Breshears et al. (2011) addressed the use of a BCI for AAC devices. This study explores BCI assistive technologies for mobility support and customized communication devices activated using tasks such as vocalization (e.g., oo, ah, eh) or tongue protrusion. These tasks can be used to control a laptop, showing how a BCI can support children with disabilities in their daily life.

#### Mental Tasks and Brain Signals

With respect to the mental tasks used in the 12 articles, motor-related tasks, P300, SSVEP, and mVEP paradigms were investigated.

Seven studies applied motor-related BCIs (Sanchez et al., 2008; Breshears et al., 2011; Pistohl et al., 2012, 2013; Taherian et al., 2017; Jochumsen et al., 2018; Zhang et al., 2019). Three studies (Breshears et al., 2011; Taherian et al., 2017; Zhang et al., 2019) applied MI paradigms. Three articles (Taherian et al., 2017; Jochumsen et al., 2018; Zhang et al., 2019) applied motor potential BCIs. There are many benefits to the use of movement-related BCI tasks (Taherian et al., 2017; Zhang et al., 2019), such as the ability to perform the task without intact visual abilities. Additionally, motor-related control may allow for control signals that are intuitive, such as imagining moving the right hand to turn a wheelchair to the right. Lastly, high achieved accuracies unlock the potential to control devices with as many degrees of freedom as physical movement. However, many researchers questioned the feasibility of MI tasks for children with congenital movement conditions, such as CP (Lust et al., 2016). The concept of MI is abstract and may be difficult for some children to understand. Furthermore, children with severe physical disabilities who have never had functional control of their limbs might find it very challenging to perform MI tasks. The included studies focusing on movement-initiated BCIs (Breshears et al., 2011; Pistohl et al., 2012, 2013; Jochumsen et al., 2018) made excellent first steps toward decoding neuronal activity related to movement. For target users who are unable to perform these movements, the BCIs must be prepared to detect the imagination of these movements for these tasks to be useful.

Five included studies (Ehlers et al., 2012; Beveridge et al., 2017, 2019; Norton et al., 2018; Vařeka, 2020) investigated the use of evoked potential paradigms (P300, SSVEP, and mVEP). One of the main benefits of using evoked-potentials for BCIs is the ability to create a system with extremely large degrees of freedom as the detection of one evoked potential allows for quick selection among several presented options. The use of mVEP (Beveridge et al., 2017, 2019) and P300 (Vařeka, 2020) is quite new in BCI research. P300 is the most successful BCI task in adult users due to its low training time to gain proficiency and high achievable accuracies (Abiri et al., 2018). P300 allows users to select among dozens of options and might be considered the best method for applications for assistive technology and AAC devices. Vařeka (2020) showed for the first time the feasibility of P300 with children reaching promising classification performance (62–79% accuracy). One of the findings of Vařeka (2020) study is the large variability of P300 components present in children's signals. This is probably due to the large age range (7–17) used as participants' inclusion criterion. Unfortunately, due to the rate of flashing options, P300 may have the potential to induce photo-epileptic seizures in users with epilepsy or in young users who are unaware of their photosensitivity. For the same reason, SSVEP tasks must also be used with caution with children with disabilities, especially for children with CP who often have visual impairments (Gabis et al., 2015). mVEPs may be a possible alternative to flash-based BCIs and have been applied in BCI spelling applications (Hong et al., 2009) and neurogaming (Beveridge et al., 2016) in adults. Beveridge et al. (2017) is the first study that applied mVEP paradigm with children and youth obtaining reasonable online performance at 70% accuracy. mVEP-based BCIs are similar to P300 BCIs as users focus on a single option among several. The mVEP paradigm relies on N200 ERPs generated when visual motion occurs on the option that the user is focusing their attention on. This paradigm may be more suitable for children who are photosensitive as it does not involve any flashing lights. Overall, these evoked tasks require immense focus and maintenance of gaze on the computer screen for the brain potentials to be evoked. This can be challenging to achieve for children with disabilities, especially for those who often have involuntary movements, such as children with CP. Additionally, evoked potential paradigms require intact sensory function. Children with CP often have visual impairments which preclude the use of these evoked tasks (Gabis et al., 2015).

An alternative to visual evoked potentials is the P300-based BCI that uses covert speech or mental singing. Both tasks have been extensively investigated in adult BCI research and should be explored with children as it requires intact hearing alone. Age-related differences in EEG responses have been explored in prior works related to auditory stimuli (Kolev and Yordanova, 1997; Sanders and Zobel, 2012). Although auditory evoked potentials are not fully mature until at least 16 years of age, research indicates that children around 5 years of age show spatially selective attention on auditory evoked potentials when listening to one of the two simultaneously presented non-verbal sounds (Sanders and Zobel, 2012). These findings are further supported by research studies (Kolev and Yordanova, 1997; Sanders and Zobel, 2012), which show clear evidence that auditory P300 signals in pediatric age should be investigated in future research.

Overall, when selecting a mental task for children, it is critical to implement tasks that are intuitive and require low effort. Ehlers et al. (2012) reported pediatric-specific deficits in the ability to perform a visual search task that the adult participants could perform. Additionally, user fatigue and visual annoyance are factors to consider when selecting a task. This systematic review revealed that only three studies (Breshears et al., 2011; Pistohl et al., 2013; Zhang et al., 2019) focused on mental tasks geared toward children and youth. Norton et al. (2018) noted that two children were visibly distracted during their calibration phase and attributed this to their choice of boring tasks. Beveridge et al. (2017, 2019) justified lower classifier performance speculating fatigue and reduction of interest or waning concentration, but they did not introduce any qualitative assessment and they did not ask participants about these factors. It is critical that engaging tasks are selected for these studies. Zhang et al. (2019) specifically focused on comparing two types of activities: a toy car and a computer cursor. They found improved BCI control when children were controlling a toy car. This may be due to the improved engagement the toy created for the participating children. Future studies should focus on creating engaging tasks to foster the optimal performance of the children and collect qualitative and quantitative evaluation factors to describe how children's performance vary along with their development.

#### Signal Acquisition Modality

Eight of the studies investigating BCIs as an access technology involved the non-invasive EEG signal acquisition modality (Ehlers et al., 2012; Beveridge et al., 2017, 2019; Taherian et al., 2017; Jochumsen et al., 2018; Norton et al., 2018; Zhang et al., 2019; Vařeka, 2020). The remaining four studies involved invasive ECoG-BCIs with children with intractable epilepsy (Sanchez et al., 2008; Breshears et al., 2011; Pistohl et al., 2012, 2013). As a result, electrode arrays were placed according to the requirements of the clinical epilepsy evaluation. Thus, not all the desired regions were included for motor task detection.

When discussing the use of ECoG-BCI research with children, it is important to consider whether brain signal acquisition is safe for a developing brain and whether there are negative long-term effects. While the meta-analysis was not an appropriate approach for this review, in general, studies involving ECoG yielded more successful outcomes than those involving EEG. This is likely due to the larger detectable frequency range, higher spatial resolution of ECoG than EEG (mm vs. cm), improved signal-to-noise ratio, and the potential to control more complex devices that require detecting small and specific patterns of brain activity.

#### EEG Signal Challenges

Main challenges faced when developing pediatric BCIs have previously been outlined by Ding et al. (2008) and Giedd et al. (1999) describing the ongoing development of a child's brain and its reorganization in presence of a brain injury and in those with atypical brain organization (Johnston, 2009; Deng, 2010; Pannek et al., 2014). EEG potentials generated by developing pediatric brains differ from adults, rendering signal features commonly extracted for successful adult BCIs potentially useless. Neurodevelopmental consequences of brain injury result in additional differences in EEG signal patterns when compared to able-bodied adults. Children's psychological and physiological state can influence the performance as the prefrontal cortex continues to rapidly develop until the age of 25 (Arain et al., 2013). Structural and functional MRI studies involving tasks used in BCI systems can be referenced to inform electrode positioning and source localization. Also, age-specific head-models are missing in EEG and MRI studies. Finally, challenges exist regarding EEG signal acquisition. High-density, gel-based, and wired EEG devices involving long training sessions are not ideal for children, especially those with disabilities. Children can experience more sensory sensitivities to gel, abrasion, and headgear. There is not a wide range of dry, active, and/or wireless headsets available for pediatric head sizes, nor for those with differences in head shape (Sellers et al., 2009; Slater et al., 2012; Hairston et al., 2014).

#### ECoG Signal Issues

Since the participants of the presented ECoG studies have intractable epilepsy and there was not a control group, it is possible their atypical brain activity contributed to identification of features applicable only in children with epilepsy (Sanchez et al., 2008; Breshears et al., 2011; Pistohl et al., 2012, 2013). Another limitation of the ECoG modality is its invasive nature and requirement of a craniotomy to implant an electrode grid (Nicolas-Alonso and Gomez-Gil, 2012). This poses significant health hazards (Nicolas-Alonso and Gomez-Gil, 2012) and creates a lack of feasibility for widespread use. Long-term stability of the signals acquired by ECoG and the longevity of the implanted grid are currently uncertain (Nicolas-Alonso and Gomez-Gil, 2012). The grids utilized in the ECoG studies were not placed for long-term use. As children grow and develop, it is unclear whether the implanted electrode grid would shift or cause damage. These considerations should be addressed in future studies on ECoG-BCI.

#### Personalized Methods

Each article presented in this review applied the same channels and features among participants included in each research study. Even when efforts are made to create a homogenous group of participants, individual differences in brain activity when performing the same task should be considered. Personalized channel and feature selection would maximize individual BCI performance. For example, a multitude of features could be extracted offline and a feature selection algorithm would then implement the top features for online use.

#### Outcome Measures

Among the reviewed studies there is inconsistency in the metrics used for presenting results as summarized in Table 7. Included studies rarely reported classification results in terms of sensitivity and specificity. Studies reported: accuracy (Sanchez et al., 2008; Breshears et al., 2011; Ehlers et al., 2012; Beveridge et al., 2017, 2019; Jochumsen et al., 2018; Norton et al., 2018; Vařeka, 2020); bit rates (Norton et al., 2018); correlation coefficients (Pistohl et al., 2012); performance score (Taherian et al., 2017); ITR (Beveridge et al., 2017, 2019); latency (Norton et al., 2018; Beveridge et al., 2019); true positive and false positive ratios (Pistohl et al., 2013); Cohen's Kappa score (Zhang et al., 2019); precision, recall and AUC (Vařeka, 2020). How much the decoding accuracy deviates from chance level should be always reported. Chance level refers to the rate achieved by random classification. For a 2-class problem, the theoretical chance level is 50%, for a 5-class problem it is 20%, etc. Unfortunately, the theoretical chance level is valid only for a large number of samples (or trials). We noted that the chance level used by most of the studies was based on the theoretical level of chance (Breshears et al., 2011; Pistohl et al., 2012; Beveridge et al., 2017, 2019). Zhang et al. (2019) considered a 70% chance level based on previous studies with adult participants (Scherer et al., 2013; Jeunet et al., 2016). The chance level of a BCI system created from a relatively small data set depends on the number of classes, sample size, and threshold for statistical significance of the classification (Combrisson and Jerbi, 2015). A simple binomial distribution involving these variables generates the threshold that must be surpassed for statistically significant classification accuracies (Combrisson and Jerbi, 2015). Another method for determining statistical significance of results is through the permutation test (Nichols and Holmes, 2002; Good, 2013).

Sanchez et al. (2008), Ehlers et al. (2012), Pistohl et al. (2013), Taherian et al. (2017), Norton et al. (2018), and Vařeka (2020) did not report the chance level. Jochumsen et al. (2018) is the only included study that estimated the chance level based on the number of trials (Müller-Putz et al., 2008).

Furthermore, for applications involving mobility, timing, and precision of the BCI output are of utmost importance. If the output were controls for a wheelchair, imprecise movements or timing delays could result in dangerous consequences for the user. Future research studies should consider reporting the same metrics at least in terms of accuracy, sensitivity, specificity, latency, and bit rates.

### Future Research and Requirements for Clinical Translation

Although much of what we understand about BCI for communication and mobility has been gained by exploring the responses of able-bodied adults to various brain activity protocols, research into BCI for individuals with disabilities has been increasing in recent years. Despite this, few research studies address the application of BCIs for communication and mobility in children. BCIs hold the potential to enable people with severe physical disabilities, who are unable to speak and who do not have voluntary muscle control, to communicate and operate other technologies without any physical movements (Wolpaw et al., 2002). A small number of studies provide encouraging evidence for continued research in children with studies demonstrating children as young as 7 years of age (Ehlers et al., 2012; Zhang et al., 2019) can learn to control their brain activity and perform activities on the computer. Recently, there has been an emergence of two new mental tasks being investigated with children (mVEP and P300) and a study that recruited unprecedented numbers of children (Vařeka, 2020). However, the clinical translation of pediatric BCIs still requires additional research to address several challenges. To summarize our recommendations, we report a list of potential requirements to be considered for BCI clinical translation. Future studies should:

- Continue to recruit large numbers of participants akin to Vařeka (2020).
- Collect smaller sub-groups of child age ranges, instead of large age ranges (e.g., 7–17), to investigate as neurodevelopment varies across childhood-adolescence.
- Include comparisons between children and adults.
- Include a TD group as a control, for studies focused on children with disabilities.
- Report the age and sex information, as their correlation with BCI performance would result in a better understanding of how to customize BCIs for children across stages of neurodevelopment.
- Collect more accompanying qualitative data, considering physiological factors, BCI experience, fatigue, and workload through standardized questionnaires to avoid bias.
- Develop study protocols specific for children and include additional training phases for those who do not have experience with BCIs.
- Increase preparation time and reduce the participant discomfort during data collection. An additional requirement is the comfort of wearable headsets and caps. Children are more sensitive to fatigue and discomfort, therefore wearing portable headsets for a long period of time can be challenging. Novel headsets specifically designed for children's heads should be developed.
- Include game activities. Considering the difficulty of children to maintain attention during mental tasks, BCI paradigms should be engaging and include customized activities and games. Also, games similar to those successfully implemented in the ADHD BCI studies could be utilized in future studies as pediatric appropriate activities. These reward-based attention games could be employed as training for BCI sessions to maintain focus as BCI sessions also require intense engagement of children through the presentation of cues and stimuli (Gavin and Davies, 2007).
- Develop customizable BCI algorithms and investigate the best algorithms suitable for pediatric BCIs. As highlighted in this systematic review, there is still no feature extraction or machine learning technique clearly established as state-of-the-art for pediatric BCIs.
- Improve performance accuracy in predicting the user's intent (Wolpaw and Wolpaw, 2012) especially in children, as they historically achieve inadequate performance for practical use (Mikołajewska and Mikołajewski, 2014).
- Be consistent in reporting algorithm performance results. In the case of binary classification, results should be reported in terms of accuracy and sensitivity (e.g., classifier sensitivity, specificity, standard deviation, etc.). In multi-class paradigms, other performance metrics should be considered like recall, precision, and F1-score. In this case, accuracy may be difficult to interpret, especially if unbalanced classification (i.e., different number of samples per class).
- Share datasets and frameworks. In fact, we noticed that none of the publications mentioned the possibility of sharing children's brain signals or sharing their code or framework, except the study by Vařeka (2020) that used a public dataset (Mouček et al., 2019) and shared the software code used for reproducibility of the obtained results. Future studies should consider publishing their datasets and sharing data analysis frameworks to better understand the feasibility of introducing BCI technology in clinical practice.
- Investigate new technologies combining multiple brain signals in pediatric populations. For example, hybrid BCI systems (e.g., fNIRS-EEG BCIs) could achieve better performance accuracy rates than single modality systems (Wolpaw and Wolpaw, 2012; Sereshkeh et al., 2019).
- The error-related potential (ErrP) can be detected in the EEG if a person perceives the mistake. Two components of the ErrP can be identified: the error-related negativity (ERN or Ne) which is a negative potential peaking 50–100 ms after an erroneous response, and the error-related positive potential, called error positivity (Pe), follows the ERN. Several studies examined ErrP in adults but only a few studies have investigated ErrP in children. Also, results are not consistent among these studies. ERN may be reliably detected before age 12 (Davies et al., 2004), or it may be present at 10 years of age (Santesso et al., 2006), or even at 7–8 years of age (Kim et al., 2007; Wiersema et al., 2007). The amplitude was found to be smaller for children compared to adults (Wiersema et al., 2007) but no significant differences were found (Kim et al., 2007). Furthermore, ERN can even be reliably elicited in children as young as 5–7 years old (Torpey et al., 2009). As such, research on ErrP should investigate if ErrPs are clearly present in pediatric brain signals.
- Prioritize BCI research with pediatric participants to better understand if a BCI system can be used as assistive technologies with children.

### Limitations

Substantial heterogeneity in the included studies concerning the participant's diagnosis, age, tasks, methods, and outcome measure prevented any pooling or meta-analysis of results for this systematic review. Our conclusions may be influenced by the small sample sizes of the identified studies that included only case studies or small groups of pediatric participants. We excluded studies aimed at providing diagnoses or therapeutic interventions in this systematic review because we targeted communication and mobility BCIs. Also, the focus on English-written articles forms a limitation of this study as other languages were not captured. Our decision to appraise the quality of included studies using QualSyst may create limitations. The checklist items represent the authors' perception of research quality and given the absence of gold standard BCI protocols, it is difficult to accurately assess the validity of the tool itself, but it was the only one applicable to the study design of the 12 articles. Finally, the use of summary scores to categorize studies may introduce bias into a review (Kmet et al., 2004). It is important to note the two research papers, Lim et al. (2012) and Rohani et al. (2014), fall outside our inclusion criteria because they focused on improving attention in children with attention-deficit and hyperactivity disorders (ADHD). They found successful results using EEG BCI-based games. Furthermore, to highlight the importance of this topic, we highlight that 10 papers screened were excluded only because they did not report BCI performance (e.g., Antle et al., 2018; Park et al., 2019).

## Conclusion

This systematic review presented the state-of-the-art of BCIs for children. It highlighted previously successful methods and paradigms and outlined actions that should be taken to develop new access technologies based on brain activity for children with severe disabilities. Despite very few research studies addressing the application of BCIs for communication and mobility in children, results are encouraging, and future research should investigate how BCIs can be better customized for pediatric ages.

## Data Availability Statement

The data supporting this systematic review are from previously reported studies and datasets, which have been cited. The processed data used to generate tables and figures of this manuscript are available from the corresponding author upon request.

## Author Contributions

SO and SCH performed data search, data analysis, and wrote the manuscript in consultation with PK, RS, and TC. SO, SCH, PK, and RS performed article identification. Article's design, data acquisition, and analysis of content were made by consensus among all the authors. TC supervised the project.

## Conflict of Interest

The authors declare that the research was conducted in the absence of any commercial or financial relationships that could be construed as a potential conflict of interest.
